# Signed Total Hip–Femoral Neck T-Score Discordance and Prevalent Proximal Femur Fracture in Elderly Hip-DXA Patients

**DOI:** 10.3390/medicina62091662

**Published:** 2026-08-29

**Authors:** Seung-Gil Jeong, Jaemin Cho, Ju-Yeong Kim

**Affiliations:** 1 Graduate School of Data Science, Pusan National University, 2 Busandaehak-ro 63beon-gil, Geumjeong-gu, Busan 46241, Republic of Korea; gil@pusan.ac.kr; 2 Department of Orthopedic Surgery, Gyeongsang National University Changwon Hospital, Gyeongsang National University School of Medicine, 11 Samjeongja-ro, Seongsan-gu, Changwon 51472, Republic of Korea

**Keywords:** bone mineral density, DXA, T-score discordance, femoral neck, total hip, proximal femur fracture, prevalent fracture, fracture association, discrimination, restricted cubic spline

## Abstract

*Background and Objectives*: Total hip (TH) and femoral neck (FN) T-scores from the same dual-energy X-ray absorptiometry (DXA) examination often diverge. We evaluated whether the signed TH–FN difference is associated with prevalent proximal femur fracture and whether it has individual-level discriminative value, explicitly separating statistical association from discrimination. *Materials and Methods*: In a single-center retrospective case–control study (complete case N = 3752; 448 fractures), multivariable logistic regression adjusting for the TH T-score and 12 covariates estimated the association of the signed difference, interaction with severity, and standalone and incremental discrimination (AUC, cross-validated ΔAUC). Additional analyses examined standardized and BMD-scale exposures, a low-energy-restricted cohort, a subtype case–case comparison, and adjustment for the ISCD lowest-site T-score including the lumbar spine. *Results*: The signed difference was associated with fracture (adjusted OR 1.65, 95% CI 1.39–1.97; interaction OR 1.06, 0.75–1.50), persisting with inpatient-restricted controls (OR 1.74), standardized and BMD-scale exposures, and low-energy restriction (OR 1.68). Standalone discrimination was weak (AUC 0.547) and out-of-sample incremental value negligible (ΔAUC +0.005, 95% CI −0.009 to +0.014). Adjusting for the ISCD lowest-site T-score abolished the full-cohort association (OR 1.04, 0.88–1.23)—the adjusted signal is femoral neck information already captured by the lowest-site rule—while a residual association persisted within the TH ≤ −2.5 subgroup (OR 1.34, 1.02–1.76). Fracture proportions are within-sample case–control fractions, not population incidence. *Conclusions*: The signed TH–FN discordance is a robust, routinely derivable correlate of prevalent proximal femur fracture whose standalone discrimination is nonetheless limited—a concrete demonstration that statistical significance need not confer predictive utility. It is best positioned as a supplementary, hypothesis-generating marker; any clinical role requires prospective validation against FRAX and the ISCD lowest-site rule.

## 1. Introduction

Hip and femur fractures remain among the most clinically devastating consequences of osteoporosis in the elderly [1]. Globally, the annual incidence of hip fractures exceeds 1.6 million and is projected to rise to 6.3 million by 2050 as populations age [2]. In South Korea, the age-standardized hip fracture incidence has risen substantially over the past two decades, with one-year mortality following hip fracture estimated at 15–20% [3,4]. Beyond mortality, hip fractures impose enormous burdens on physical function, independence, and healthcare costs, making the accurate identification of skeletal fragility a public health priority [5,6,7].

Dual-energy X-ray absorptiometry (DXA) of the hip is the reference standard for bone mineral density (BMD) measurement and osteoporosis diagnosis [8,9], and low hip BMD is among the strongest predictors of subsequent fracture [10]. Per WHO criteria, osteoporosis is defined as a T-score ≤ −2.5 SD below the young-adult mean at the TH or FN region of interest [11]. The International Society for Clinical Densitometry (ISCD) recommends diagnosing osteoporosis using the lowest T-score from either site, acknowledging that TH and FN T-scores often diverge [12,13].

This site-specific divergence—termed T-score discordance—arises because the TH region encompasses a weighted average of subregions (femoral neck, trochanter, and intertrochanteric), while the FN T-score reflects areal BMD over the smaller femoral neck region alone (cortical and trabecular bone). Discordance between skeletal measurement sites—classically between the hip and the spine—has been documented in a substantial fraction of clinical DXA examinations [14,15].

Prior studies have largely characterized the *prevalence and determinants* of inter-site discordance [14,16,17]. A separate literature has shown that regional proximal femur BMD differs by hip fracture type, with intertrochanteric fractures generally showing lower trochanteric and intertrochanteric BMD than femoral neck fractures, with site-matched BMD best distinguishing each fracture type [18,19,20,21]; that a signed inter-site contrast (low trochanteric with relatively preserved femoral neck BMD) can independently discriminate fracture type [22]; and that a localized femoral T-score difference (femoral neck versus trochanter) varies across hip-fracture sites [23]. Yet the way in which the directional TH–FN difference relates to prevalent fracture in a fully available hip-DXA population—and whether it adds discriminative value—remains unclear. Rather than presuming that discordance predicts fracture, we approached the question empirically: we examined the signed differences among the four hip ROIs (TH, FN, trochanter, and intertrochanteric) and focused on the TH–FN difference. Critically, evaluating such a marker requires distinguishing *statistical association* from *individual-level prediction*—in a large sample, even a weak association reaches significance, but statistical significance does not imply discriminative or predictive utility.

Accordingly, we posed four questions: (i) Is the signed TH–FN difference associated with proximal femur fracture? (ii) Does the relative magnitude of that association *vary with T-score severity* (effect modification)? (iii) Can the difference *predict* fracture on its own, and does it add incremental information beyond TH severity? (iv) In which population does the absolute fracture rate separation become large enough to be clinically noteworthy? To address these, we used the fully available elderly hip-DXA cohort as the primary analysis—rather than restricting to osteoporotic patients—and adjusted for TH T-score directly. Meanwhile, we formally tested interaction, quantifying discrimination (AUC), and incremental value, and examined absolute fracture rate separation; a pre-specified osteoporosis subgroup and a dual-criterion cohort framed the clinical application context.

## 2. Materials and Methods

### 2.1. Study Design and Participants

This was a single-center retrospective case–control study conducted at Gyeongsang National University Changwon Hospital, Changwon, Republic of Korea, spanning January 2016 to December 2025, and is reported in accordance with the STROBE guidance for observational studies [24]. The institutional review board granted an exemption from review (approval no. GNUCH 2026-07-022), and individual informed consent was waived given the retrospective design and use of de-identified data. Cases were patients aged ≥65 years admitted with a proximal femur fracture during the study period and whose qualifying hip DXA was obtained within 2 weeks of the index fracture. Bone mineral density is unlikely to change biologically over so short an interval, but the measurement represents peri-fracture rather than prospective baseline status. In fracture cases the qualifying DXA was obtained before surgical treatment and was measured on the contralateral (non-fractured) hip, avoiding acute fracture-site artefact; in controls, the measured hip was the side confirmed free of pathological conditions on clinical evaluation, in accordance with the standardized acquisition protocol. Controls were patients aged ≥65 years who underwent hip DXA at the same institution during the same period without a documented femur fracture; controls underwent DXA for routine clinical indications (osteoporosis screening or follow-up of low bone mass in outpatients, or evaluation during an unrelated admission in inpatients) and are therefore not a random population sample; controls were not individually matched, the full available control pool was retained, and confounding was addressed by multivariable adjustment. We enrolled 3788 patients (452 cases, 3336 controls; control pool: 1741 inpatients and 1595 outpatients) meeting the following criteria: (1) age ≥65 at DXA; (2) hip DXA with all four ROI measurements; (3) for cases, a qualifying hip DXA within 2 weeks of the index fracture.

### 2.2. DXA Measurements and T-Score Variables

Hip DXA was performed with standardized clinical protocols on Hologic Horizon densitometers, with T-scores referenced to young-adult normative data [25]. Four ROI T-scores were extracted: total hip (TH), femoral neck (FN), trochanter (TR), and intertrochanteric (IT), all complete. Two features of this construct should be noted: the FN region is anatomically contained within the TH region (TH is a composite of the neck, trochanter, and intertrochanteric subregions), so the TH–FN difference re-weights an already-included subregion rather than contrasting two disjoint sites. Additionally, because TH and FN T-scores are referenced to different young-adult normative databases, the difference carries a systematic inter-database offset (median ≈ 0.5 SD in this cohort), so the modeled gradient reflects patient-level discordance around that fixed offset. TH T-score and FN T-score were the clinical ROIs of interest; TR T-score and IT T-score were used only in exploratory analyses (Appendix B). The primary exposure was the signed TH–FN difference = TH T-score–FN T-score; a positive value indicates a relatively worse (lower) FN. The absolute difference (|signed difference|) was used for the binary contrast (≥1.0 vs. <1.0—a clinically interpretable 1 SD inter-site contrast chosen *a priori*: because T-scores are expressed in standard deviation units, one full SD is the natural interpretable unit of inter-site contrast—the unit in which BMD fracture gradients are conventionally expressed [26]; the continuous and spline analyses confirm that the findings do not depend on this choice—not a diagnostic cutoff); the signed difference was used for continuous and spline analyses. This 1.0 SD contrast applies to the difference between two T-scores and is conceptually distinct from the T-score value of −1.0 that defines the lower bound of normal BMD. Lumbar spine (L1–L4 total) T-scores, available from the structured DXA reports in 3731/3788 patients (98.5%; the remainder underwent hip-only DXA), were used in an additional analysis of the ISCD lowest-site rule (Section 2.7).

### 2.3. Outcome Variables

The primary outcome was any proximal femur fracture; all outcome fractures were femoral neck or intertrochanteric, and we therefore use “proximal femur fracture” throughout. Secondary outcomes were the femoral neck (n = 219) and intertrochanteric (n = 229) subtypes, classified from the structured diagnosis field with no overlap. Fracture mechanisms were predominantly low-energy falls; 52 cases followed high-energy trauma and one carried an ambiguous energy code with a documented ground-level fall, though a low-energy-restricted sensitivity analysis excluded these 53 cases (Section 2.7). A structured audit of the diagnosis and admission note text identified no pathological fractures (0/452); a documented history of malignancy at non-femoral sites was present in 13 cases, all with clearly documented fall mechanisms. Each secondary analysis comprised the relevant subtype cases plus the shared non-fracture controls, excluding the opposite subtype; because they share the control pool, the two subtype analyses are not independent and their effect sizes are not directly compared.

### 2.4. Covariates

The full cohort primary model adjusted for 13 covariates: the TH T-score, age, sex (male = 1), BMI, past femur fracture, chronic kidney disease, rheumatoid arthritis, dementia, Parkinson’s disease, smoking (3 categories: never/no [reference], former, and current), alcohol (3 categories: no drinking [reference], former, and current), prior osteoporosis treatment, and history of diabetes mellitus (PHx_DM). Because the signed difference equals TH T-score–FN T-score, holding TH T-score fixed and adding the difference is algebraically equivalent to adding FN T-score—the model asks whether, among patients sharing the same TH severity, a relatively worse FN carries higher fracture odds. Explicitly, by writing *d* = TH−FN (so that FN = TH−*d*) we find the following:

logit *P*(fracture) = *β*_0_ + *β*_1_*d* + *β*_2_ TH + *γ*^⊤^X ≡ *β*_0_ − *β*_1_ FN + (*β*_1_ + *β*_2_) TH + *γ*^⊤^X,

so the two parameterizations span exactly the same information, and the coefficient of the signed difference is identical to that of the femoral neck information beyond the total hip. No claim of a construct independent of site-specific BMD is made or implied. In the TH T-score ≤ −2.5 subgroup and the dual-criterion cohort, TH T-score was the filter condition (insufficient residual variance) and was not entered as a covariate (12 covariates). A clinical-only sensitivity model additionally omitted smoking and alcohol, testing robustness to lifestyle adjustment.

**Data cleaning.** In the source file, unmeasured height, weight, and BMI were entered as the character x/X rather than left blank. An audit of all rows for which BMI could not be computed recovered nine height–weight transpositions (the recorded raw BMI matched the transposed values; all nine were controls), corrected one leading-digit omission (row 1113: height 75 → 175 cm) and one typographical error (row 3704: weight 54.50 kg), and set four implausible and one suspect short-stature record to missing. BMI was recalculated for every row as weight/(height/100)^2^, which also reconciled two rows whose recorded BMI differed from the computed value. BMI was then genuinely missing for 35 patients; one further record had an unresolvable alcohol entry. Complete case exclusion of these 36 patients yielded the analytic cohort of N = 3752 (448 fractures). The diabetes covariate (PHx_DM) was rebuilt from the past-history and inpatient problem-list text: the previous parser matched only the literal “DM,” missing notations such as T2DM, T2D, Korean diabetes notation, diabetes, and DPN, while spuriously capturing position-coded DM-negative entries, the medication “Dmab” (denosumab), and “Admission.” A comprehensive matcher with position-sign parsing and negation/family history/prediabetes exclusion increased the positive count from 774 to 1288 (cohort and complete case N unchanged).

### 2.5. Study Cohorts

Table 1 summarizes the five analysis cohorts. The full complete case cohort (N = 3752) was the primary analysis population; adjusting for TH T-score within this full available cohort maximizes power, avoids selection on the exposure-defining variable, and forestalls cherry-picking concerns. The TH T-score ≤ −2.5 subgroup (N = 913) was a pre-specified deepening anchored to the WHO TH osteoporosis threshold and framing the clinical-application context.

### 2.6. Statistical Analyses

All analyses used Python 3.10 (statsmodels 0.14.6, scikit-learn 1.7.2, scipy 1.15.3, matplotlib3.10.8, patsy1.0.2) (*statsmodels*, scikit-learn, *scipy*, matplotlib, *patsy*), two-sided α = 0.05, a fixed seed of 42, and 2000 bootstrap resamples with function-local generators for order-independent reproducibility.

#### 2.6.1. Baseline Characteristics

Continuous variables as mean ± SD (non-normal also as median [IQR]), categorical as n (%); between-group comparisons by *t*-test or Mann–Whitney and χ^2^, with standardized mean differences (SMDs).

#### 2.6.2. Binary Difference Contrast

|difference| ≥ 1.0 vs. <1.0 by multivariable logistic regression, in the full cohort (primary), subgroup, and dual cohorts. Because the absolute contrast folds the two discordance directions together, a signed binary contrast (signed difference ≥ 1.0 vs. <1.0)—the directionally consistent version—was also fitted.

#### 2.6.3. Continuous Signed Difference

Adjusted OR per 1.0-unit increment, unadjusted and adjusted, across the three cohorts.

#### 2.6.4. Restricted Cubic Splines

A restricted cubic spline (RCS) models a continuous predictor as a smooth piecewise-cubic curve joined at fixed “knots” and constrained to be linear beyond the outermost knots, allowing the difference–fracture relationship to bend (be nonlinear) without forcing a straight line or producing unstable tails. We used a natural cubic spline (*cr*(), df = 4, with boundary knots at the cohort’s minimum and maximum and two interior knots placed by the basis at data quantiles. For the full cohort the realized knots fell at signed-difference values of −4.4, −0.3, 0.8, and 3.5) following established regression-modeling guidance [27]. A likelihood-ratio test (LRT) assessed the overall association (spline terms vs. no difference) and a second LRT assessed nonlinearity (spline vs. linear difference). Predicted probabilities are shown over the 1st–99th percentile range with 95% bands (shading α = 0.5). There are two panels: full cohort and subgroup.

#### 2.6.5. Incremental Predictive Value

Nested models in the full cohort—Base (TH T-score + 12 covariates), Model A (+ linear difference), and Model B (+ RCS difference)—were compared by LRT p (df), AIC/ΔAIC, AUC and ΔAUC, IDI, and category-free NRI (cNRI) with bootstrap CIs. Added predictive value was interpreted within established frameworks for evaluating a new marker, including reclassification metrics [28,29]. TH T-score, FN T-score, and the difference were never entered simultaneously. To guard against in-sample optimism, the out-of-sample ΔAUC was estimated by repeated stratified 5-fold cross-validation (20 repeats). Model calibration was summarized by the calibration slope and collinearity by variance inflation factors (VIFs).

#### 2.6.6. Fracture Subtypes

Binary and continuous analyses were repeated for the neck and intertrochanteric subtypes (not directly compared).

#### 2.6.7. Discrimination (Association vs. Prediction)

To distinguish statistical *association* from individual-level *prediction*, the standalone discriminative ability of the signed difference (AUC of a difference-only logistic model) was computed in each cohort and, in the full cohort, was compared against TH T-score alone and TH T-score + difference, each with 95% bootstrap CIs.

#### 2.6.8. Effect Modification (Interaction) Test

Whether the *relative* difference effect varies with severity was tested formally with difference × osteoporosis-status (TH T-score ≤ −2.5) interaction terms (unadjusted and adjusted) and a difference × TH T-score (continuous) interaction, each reported with its 95% CI.

#### 2.6.9. Sensitivity and Subgroup Analyses

Lifestyle-adjusted vs. clinical-only models; exploratory subgroups by sex and age (median split at 74), dropping within-subgroup constant covariates. 

#### 2.6.10. Osteopenia Range Confirmatory Analysis

RCS within −2.5 < TH T-score ≤ −1.0 (N = 1607), examining whether the difference signal extends beyond the osteoporosis range (exploratory).

#### 2.6.11. Inpatient-Restricted Sensitivity

Because all cases were inpatients (Section 4.8), the primary continuous and binary models were refitted with controls restricted to inpatients only (cases vs. inpatient controls; N = 2179), holding admission status approximately constant to probe source-type confounding.

#### 2.6.12. Appendix Analyses

Appendix A: FN–anchor cohort (FN T-score ≤ −2.5), in which FN–TH, FN–TR, and FN–IT were each analyzed by binary, continuous, RCS, and standalone AUC (symmetric to the TH anchor). Appendix B: the six unique ROI-pair signed differences in the full cohort. Appendix C: four machine-learning feature sets in the subgroup (5-fold CV; exploratory).

### 2.7. Additional Analyses

Five additional analyses were performed. (1) *Standardized-difference sensitivity*: each T-score was z-standardized within the cohort and the difference of z-scores was modeled per SD; because a T-score is an affine transform of areal BMD, the z-standardized T-score difference is mathematically identical to a z-standardized BMD difference and is invariant to reference-database scaling. (2) *BMD-scale sensitivity*: areal BMD (g/cm^2^) was reconstructed from each ROI’s T-score by linear calibration on the 19 patients whose reports carried measured values (aBMD_FN_ = 0.8075 + 0.0922 *T*, *R*^2^ = 0.987; aBMD_TH_ = 0.8943 + 0.1052 *T*, *R*^2^ = 0.983; leave-one-out RMSE ≤ 0.018 g/cm^2^), measured values being used where available, an analysis which is supportive only. (3) *Low-energy restriction*: high energy trauma cases were excluded. (4) *Subtype case–case comparison*: among fracture cases, femoral neck versus intertrochanteric subtype was modeled directly by logistic regression. (5) *ISCD lowest-site adjustment*: in the 3696 complete case patients with lumbar T-scores, we adjusted for *T*_min_ = min(*T*_lumbar_, *T*_FN_, *T*_TH_)—the quantity used by the ISCD lowest-site rule—in place of the TH T-score, testing by likelihood ratio whether the signed difference retains information beyond the lowest-site T-score.

## 3. Results

### 3.1. Participant Characteristics

**Figure 1:**
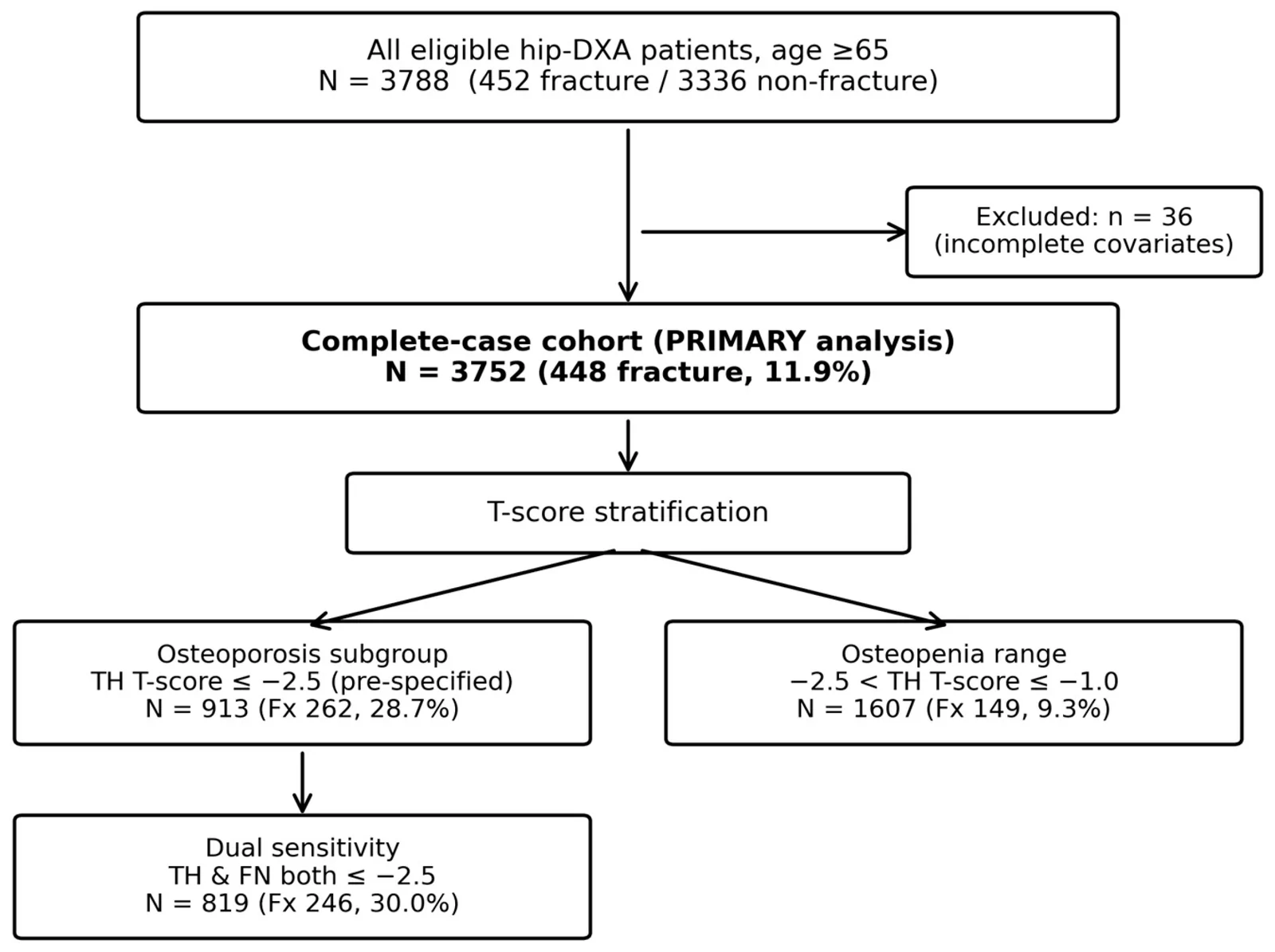
Study cohort flowchart.

Figure 1 presents the cohort flowchart. From 3788 eligible patients (452 fracture, 3336 non-fracture), 36 were excluded by complete case criteria (35 missing BMI, 1 unresolvable alcohol entry), yielding the primary analysis cohort of N = 3752 (448 fractures, 11.9%). Within this cohort, the pre-specified TH T-score ≤ −2.5 subgroup comprised 913 patients (262 fractures, 28.7%), the dual-sensitivity cohort 819 patients (246 fractures, 30.0%), and the osteopenia confirmatory range 1607 patients (149 fractures, 9.3%).

Fracture patients were substantially older (81.3 ± 7.1 vs. 73.6 ± 6.6 years; SMD = 1.116), had lower BMI (median 21.6 vs. 23.8 kg/m^2^; SMD = −0.622), and had more severe T-scores at every hip ROI (TH T-score: −2.6 ±1.2 vs. −1.4 ±1.3; FN T-score: −3.2 ±1.3 vs. −1.8 ± 1.3) (Table 2). The signed TH–FN difference was modestly higher in fracture cases (median 0.6 vs. 0.5; SMD = 0.136; *p* = 0.001)—a small absolute difference that nonetheless foreshadows the weak standalone separation quantified in Section 3.7. Dementia (20.5% vs. 4.3%; SMD = 0.509) and Parkinson’s disease (3.8% vs. 1.3%; SMD = 0.156) were more prevalent among fracture cases, whereas prior osteoporosis treatment was *less* common in cases (6.0% vs. 10.4%; SMD = −0.158)—the expected direction for a pre-baseline prescription field (Section 2.4) and the reason it, rather than a post-fracture treatment flag, was used as the covariate. By design, all fracture cases were inpatients, whereas controls were 52.4% inpatient (SMD = 1.348); this near-separation is addressed in the Discussion.

**Table 2:** Baseline characteristics by fracture status (complete case cohort, N = 3752). Continuous variables as mean ± SD or median [IQR]; categorical as n (%). SMD, standardized mean difference.

	Non-Fracture			
Variable	(n = 3304)	Fracture (n = 448)	SMD	*p*-Value
Age (years)	73.6 ± 6.6	81.3 ± 7.1	1.116	<0.001
Height (cm)	156.6 ± 8.3	157.3 ± 8.6	0.084	0.101
Weight (kg)	58.9 ± 10.5	54.1 ± 10.5	−0.454	<0.001
BMI (kg/m^2^)	23.8 [21.5–26.2]	21.6 [19.2–23.7]	−0.622	<0.001
TH T-score	−1.4 ± 1.3	−2.6 ± 1.2	−1.018	<0.001
FN T-score	−1.8 ± 1.3	−3.2 ± 1.3	−1.060	<0.001
TR T-score	−1.6 ± 1.1	−2.6 ± 1.0	−0.958	<0.001
IT T-score	−1.4 ± 1.0	−2.5 ± 0.9	−1.035	<0.001
Signed difference (TH–FN)	0.5 [0.0–0.9]	0.6 [0.1–1.0]	0.136	0.001
Male sex	955 (28.9%)	126 (28.1%)	−0.017	0.775
Past femur fracture	373 (11.3%)	73 (16.3%)	0.145	0.003
CKD	326 (9.9%)	55 (12.3%)	0.077	0.133
RA	65 (2.0%)	1 (0.2%)	−0.168	0.015
Dementia	141 (4.3%)	92 (20.5%)	0.509	<0.001
Parkinson’s disease	44 (1.3%)	17 (3.8%)	0.156	<0.001
Osteoporosis treatment (prior)	342 (10.4%)	27 (6.0%)	−0.158	0.005
PHx DM	1134 (34.3%)	145 (32.4%)	−0.041	0.443
Smoking—former	279 (8.4%)	38 (8.5%)	0.001	1.000
Smoking—current	151 (4.6%)	31 (6.9%)	0.101	0.040
Alcohol—former	171 (5.2%)	28 (6.2%)	0.046	0.401
Alcohol—current	259 (7.8%)	45 (10.0%)	0.077	0.130
Inpatient (source)	1731 (52.4%)	448 (100.0%)	1.348	<0.001

**Figure 2:**
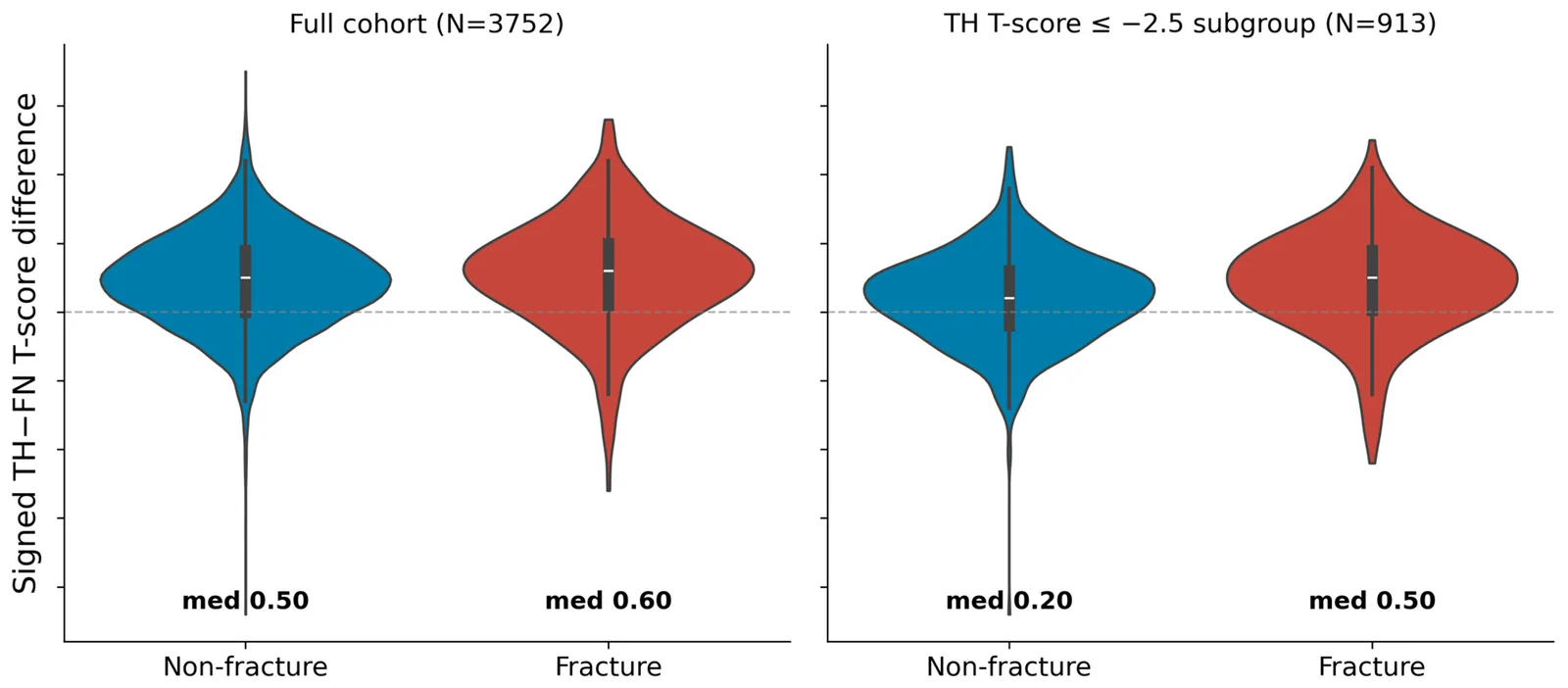
Signed TH–FN difference distribution by fracture status (full cohort and osteoporosis sub- group).

Figure 2 shows the signed TH–FN difference distribution by fracture status for the full cohort and the osteoporosis subgroup. In both panels, fracture patients showed a modest rightward shift, consistent with a higher proportion of positive (FN-worse-than-TH) discordance in the fracture group; the substantial overlap between groups previews the limited discriminative ability of the difference as a standalone marker.

### 3.2. Binary Absolute Difference Analysis

**Figure 3:**
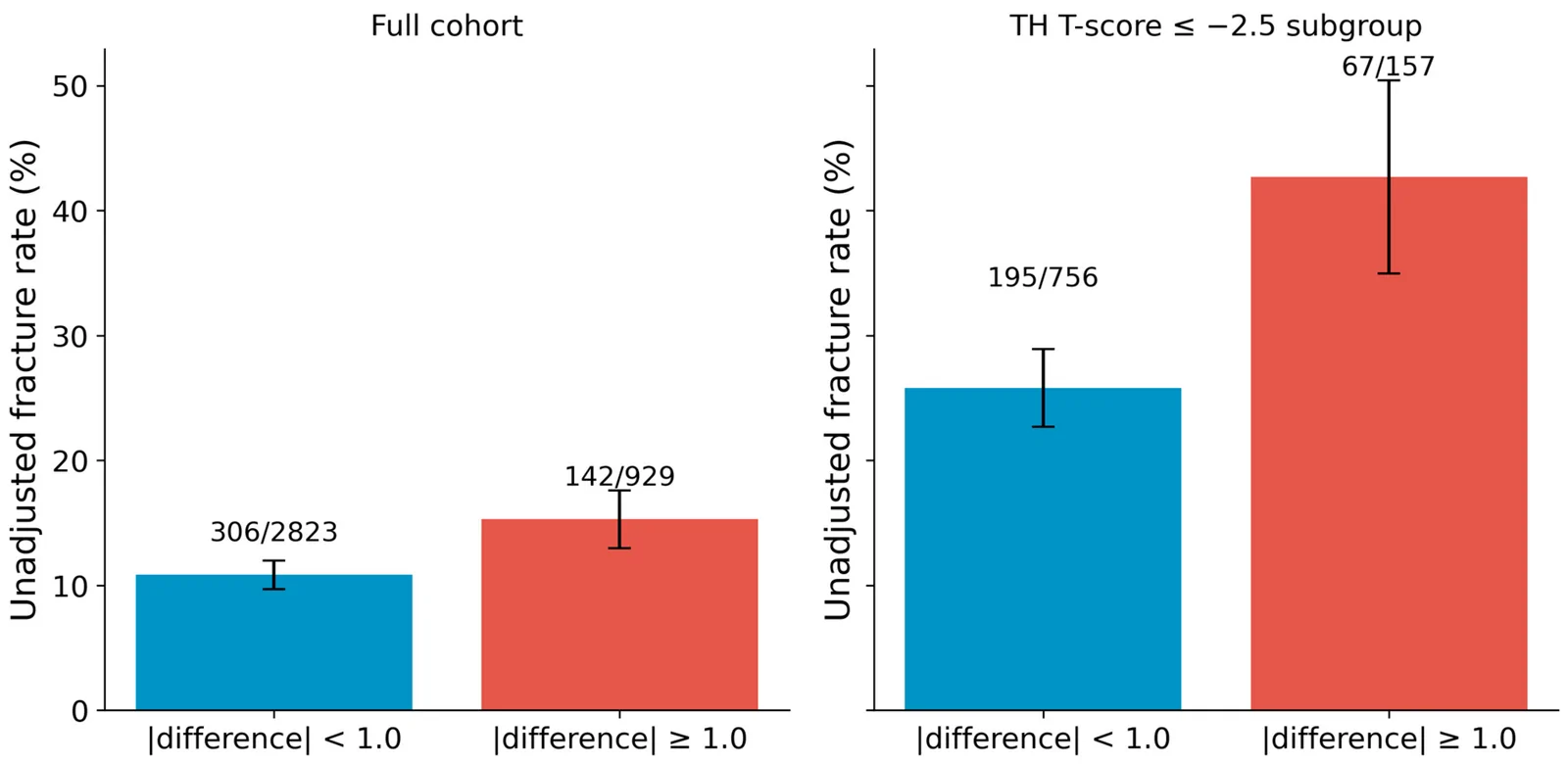
Unadjusted fracture rates by binary difference group (full cohort and osteoporosis sub- group). Rates reflect the present case–control sample (within-sample), not population incidence.

In the full cohort, 929 patients had |difference| ≥ 1.0, with unadjusted fracture rates of 15.3% vs. 10.8% in the low-difference group—a difference of 4.5 percentage points. The adjusted OR for |difference| ≥ 1.0 was 2.02 (95% CI: 1.57–2.62; *p* < 0.001) (Table 3). In the osteoporosis subgroup, the *relative* effect was similar (OR 1.86, 95% CI: 1.24–2.79; *p* = 0.003), but the *absolute* fracture rate contrast was far larger (42.7% vs. 25.8%; a 16.9 percentage-point difference). The dual-sensitivity cohort gave a concordant estimate (44.4% vs. 27.3%; OR 1.92, 95% CI: 1.24–2.97; *p* = 0.004). The widening of the absolute contrast (4.5 →16.9 percentage points) alongside a near-constant odds ratio reflects the higher baseline fracture rate of the osteoporosis range rather than a stronger relative effect; this distinction is examined formally in Section 3.8. Figure 3 displays the unadjusted fracture rates by binary difference group. We re-emphasize that |difference| ≥ 1.0 is a clinically interpretable 1 SD inter-site contrast chosen *a priori*, not a diagnostic or treatment cutoff. Because the absolute contrast combines both discordance directions, we also fitted the directionally consistent signed contrast (signed difference ≥ 1.0 vs. <1.0): the adjusted OR was 2.06 (95% CI: 1.58–2.70) in the full cohort, 1.98 (1.27–3.11) in the subgroup, and 2.03 (1.29–3.20) in the dual cohort—concordant with, and slightly cleaner than, the absolute-difference estimates. Finally, the absolute fracture rates and percentage-point contrasts reported here reflect the case–control sampling frame and should be read as within-sample, not population incidence (Section 4.8).

**Table 3:** Binary absolute-difference analysis across cohorts (high vs. low difference, threshold 1.0 SD). OR: odds ratio adjusted for the full covariate set.

Cohort	n (|diff| < 1.0)	Fx %	n (|diff| ≥ 1.0)	Fx %	Adjusted OR (95% CI)	*p*
Full cohort	2823	10.8	929	15.3	2.02 (1.57–2.62)	<0.001
TH T-score ≤ −2.5 subgroup	756	25.8	157	42.7	1.86 (1.24–2.79)	0.003
Dual sensitivity	686	27.3	133	44.4	1.92 (1.24–2.97)	0.004

### 3.3. Continuous Signed Difference Analysis

**Figure 4:**
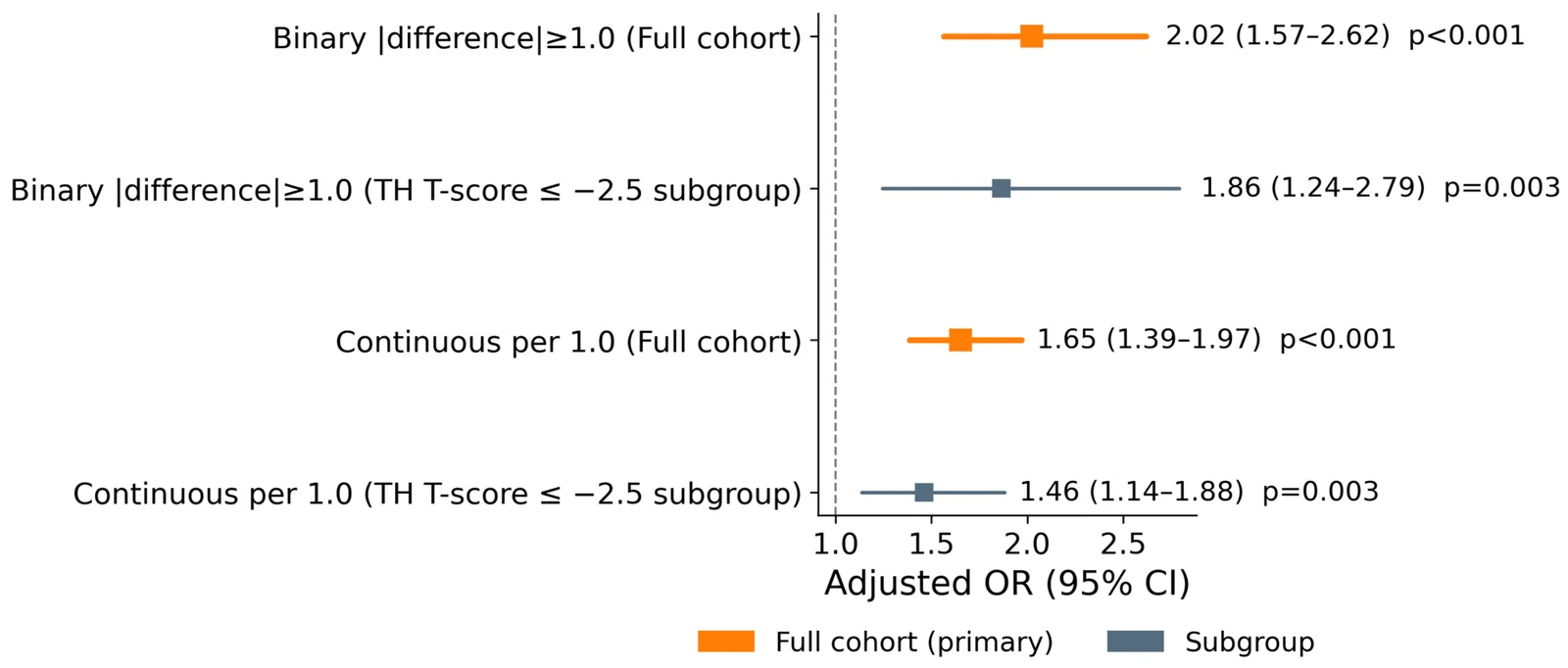
Forest plot of adjusted ORs (binary and continuous difference; full cohort and osteoporosis subgroup).

In the full cohort, each 1.0-unit increase in the signed TH–FN difference carried an unadjusted OR of 1.23 (95% CI: 1.07–1.42) and an adjusted OR of 1.65 (95% CI: 1.39–1.97; *p* < 0.001) (Table 4). Importantly, the *adjusted* estimate exceeded the *unadjusted* one: in the full cohort the difference reaches its full magnitude only after conditioning on TH T-score, because holding TH severity fixed while adding the difference is algebraically equivalent to adding FN T-score—i.e., the signal is FN information beyond TH, not a marginal property of the difference itself. The pre-specified osteoporosis subgroup yielded a comparable adjusted OR of 1.46 (95% CI: 1.14–1.88; *p* = 0.003), and the dual-sensitivity cohort 1.73 (95% CI: 1.28–2.33; *p* < 0.001). The relative association is thus consistent (adjusted OR ≈ 1.5–1.7) across the full cohort, the osteoporosis range, and the dual-criterion cohort. Figure 4 summarizes the binary and continuous estimates in a forest plot; all adjusted ORs exceeded 1.0 with 95% CIs excluding unity.

**Table 4:** Continuous signed TH–FN difference analysis (odds ratio per 1.0-unit increase) across cohorts.

Cohort	N	Events	Unadjusted OR (95% CI)	Adjusted OR (95% CI)	*p*-Value
Full cohort	3752	448	1.23 (1.07–1.42)	1.65 (1.39–1.97)	<0.001
TH T-score ≤ −2.5 subgroup	913	262	1.68 (1.35–2.10)	1.46 (1.14–1.88)	0.003
Dual sensitivity	819	246	1.78 (1.37–2.31)	1.73 (1.28–2.33)	<0.001

### 3.4. Restricted Cubic Spline Analysis

**Figure 5:**
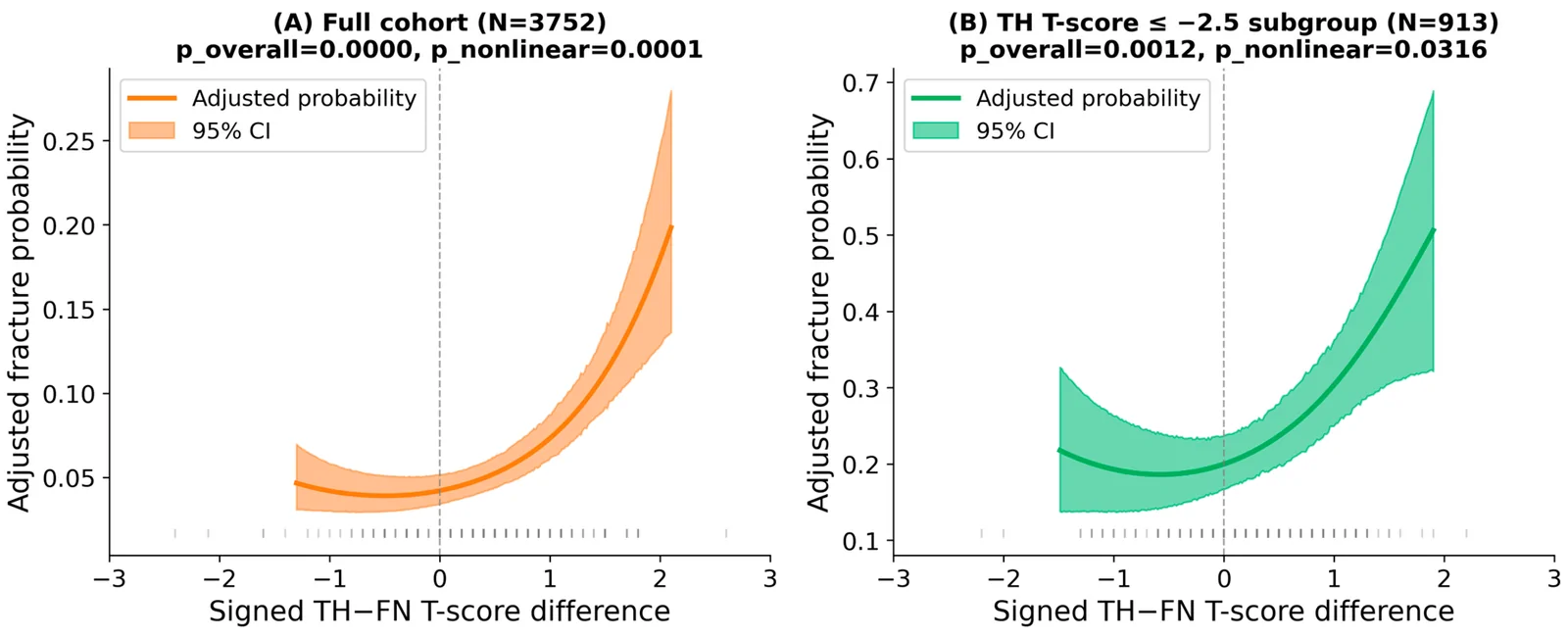
Restricted cubic spline of adjusted fracture probability versus signed TH–FN difference. (**A**) full cohort; (**B**) osteoporosis subgroup; CI shading α = 0.5. Model-predicted probabilities reflect the present case–control sample and should not be read as population incidence.

Figure 5 displays the two-panel RCS-predicted adjusted fracture probability across the signed TH–FN difference range. In the full cohort (Figure 5A, N = 3752, 448 events), the overall association was highly significant (LRT *p*_overall < 0.001) with a detectable nonlinear component (*p*_nonlinear < 0.001); predicted probability rose progressively across the difference range, steepest in the positive-difference region. In the osteoporosis subgroup (Figure 5B, N = 913, 262 events), the overall association remained significant (*p*_overall < 0.001) with a borderline nonlinear term (*p*_nonlinear = 0.032). The spline confirms an independent, monotonically increasing difference–fracture association across the T-score spectrum; consistent with Section 3.7, this association is statistically robust but spans only a narrow band of absolute predicted probability.

### 3.5. Quintile Sensitivity Analysis

**Figure A4:**
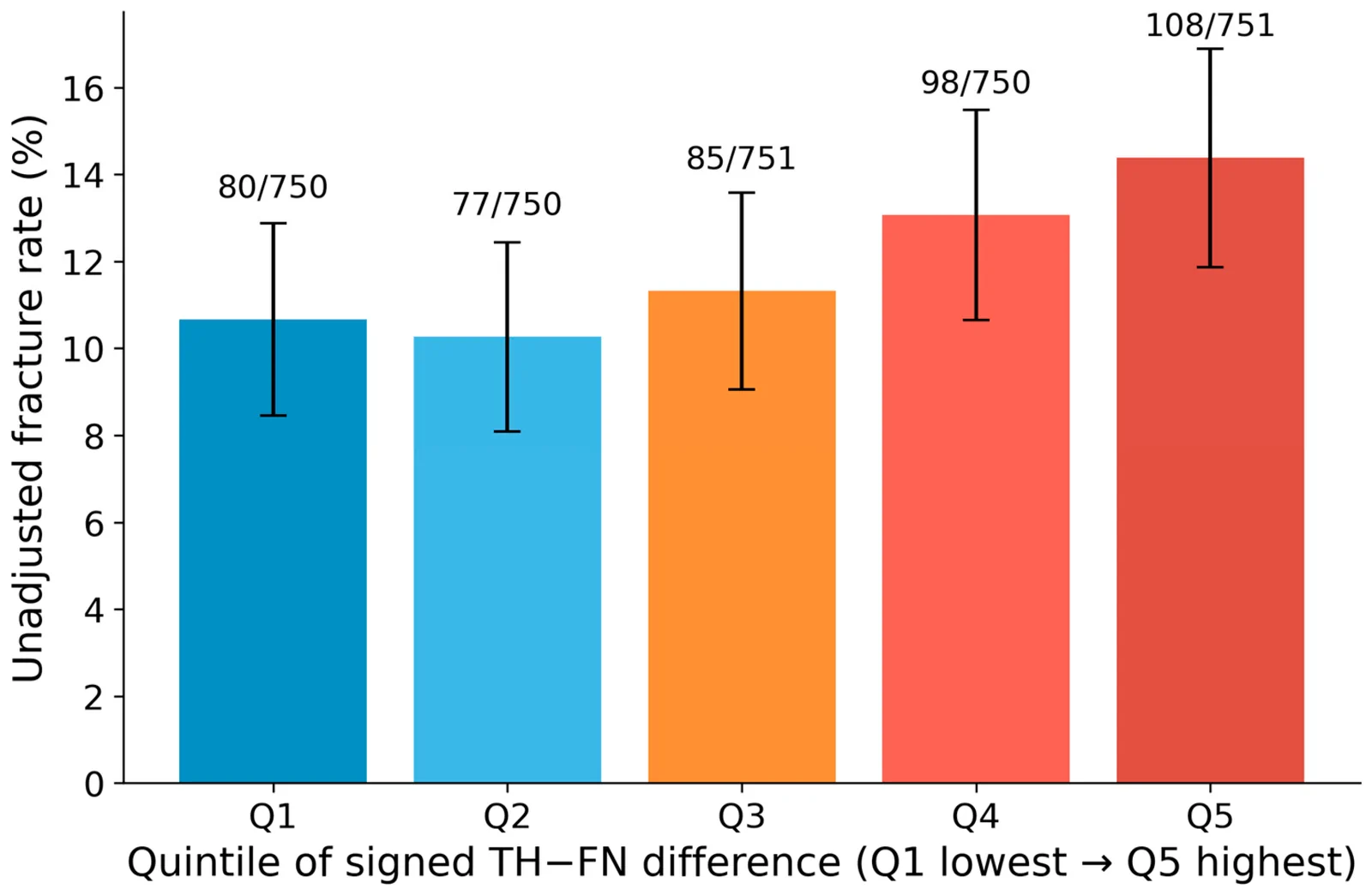
Unadjusted fracture rate by signed-difference quintile (full cohort). Rates are within- sample (case–control), not population incidence.

The equal-N quintile analysis is presented in Appendix D (Table A5, Figure A4). In brief, unadjusted fracture rates rose monotonically from 10.7% in the lowest to 14.4% in the highest signed-difference quintile, with the increase concentrated in the upper two quintiles—consistent with the spline gradient of Section 3.4—while the modest absolute spread again illustrates the limited standalone separation.

### 3.6. Incremental Predictive Value

**Figure 6:**
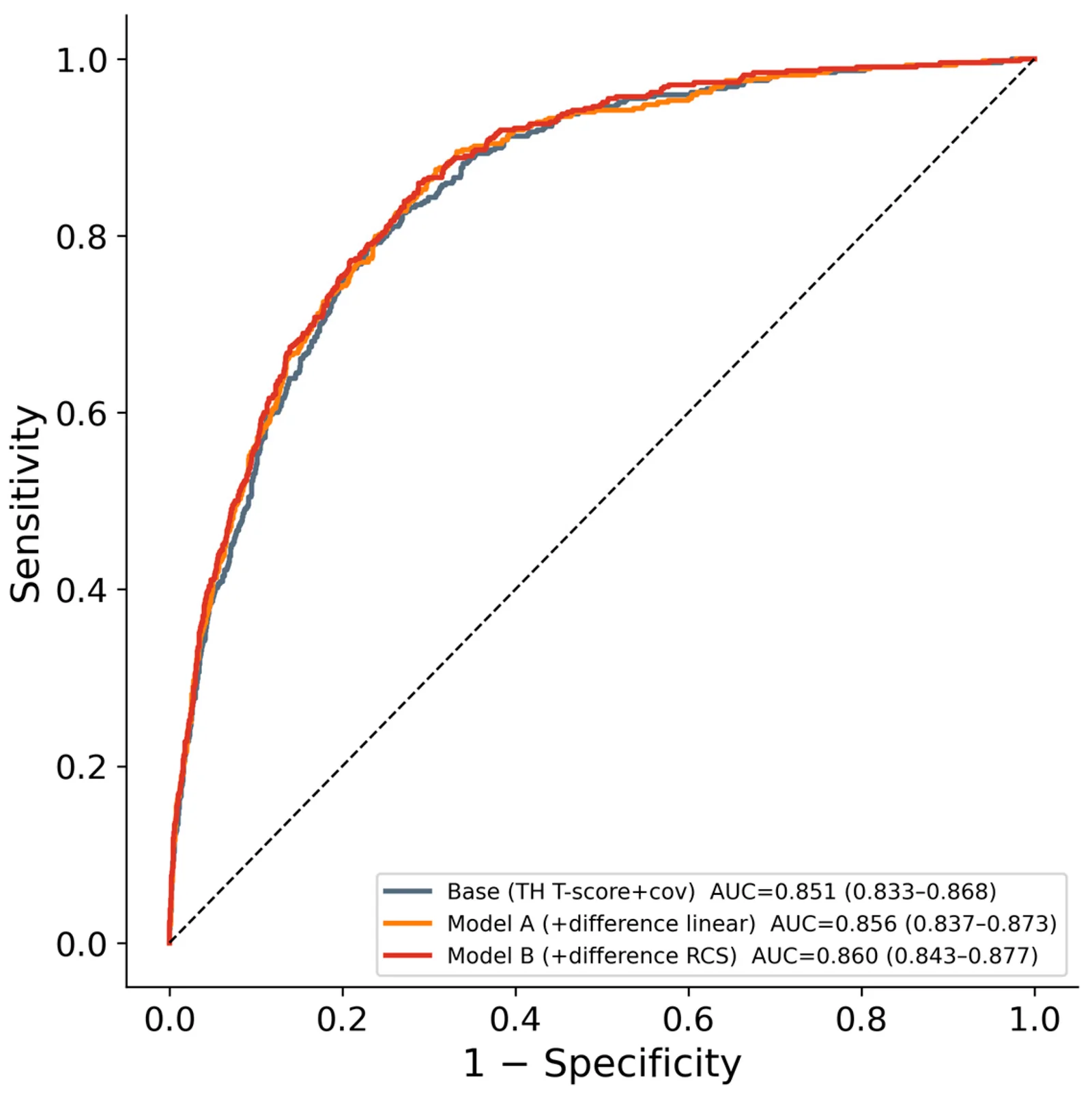
ROC curves for Base, Model A, and Model B (full cohort).

Adding the signed difference improved model fit beyond TH severity for both parameterizations (LRT *p* < 0.001 for Model A and Model B), and AIC fell by 31 (linear) and 46 (RCS) relative to base (Table 5). However, the gain in discrimination was small: ΔAUC was +0.005 (linear) and +0.009 (RCS), and the IDI was 0.0139 (95% CI: 0.0077–0.0199) and 0.0203 (95% CI: 0.0129–0.0279), respectively—statistically supported but modest separation of event and non-event predicted probabilities. The cNRI was 0.301 (95% CI: 0.204–0.400; Model A) and 0.255 (95% CI: 0.157–0.354; Model B), representing the sum of net upward reclassification in fracture cases and net downward reclassification in controls per Pencina et al. (2008) [28]; its bootstrap CI reflects re-prediction uncertainty under fixed coefficients rather than model-selection uncertainty. Because IDI and cNRI are sensitive to outcome prevalence, under the present case–control sampling they should be read as within-sample reclassification descriptors rather than population-calibrated quantities; the prevalence-invariant ΔAUC (above) carries the primary incremental value inference. The pattern—clearly significant likelihood-ratio improvement but only a few thousandths of AUC—is exactly what is expected when a single additive marker is added to a model already containing TH T-score and 12 covariates, and it motivates the explicit association-versus-prediction analysis in Section 3.7. Critically, this small increment was not an in-sample optimism artifact: under repeated stratified 5-fold cross-validation (20 repeats), the out-of-sample ΔAUC for Model A was +0.005 (95% across folds: −0.009 to +0.014, i.e., including zero), matching the apparent estimate; the Model A calibration slope was 1.00; and the variance inflation factors for TH T-score and the difference were both 1.05, indicating that the difference is essentially orthogonal to (not collinear with) TH severity. The honest reading is therefore that the incremental discrimination is real in fit but negligible and not demonstrably non-zero out-of-sample. Figure 6 shows the overlapping ROC curves.

**Table 5:** Incremental predictive value of the signed difference across three nested models (full cohort). Base = TH T-score + 12 covariates; Model A = Base + linear difference; Model B = Base + restricted cubic spline difference (df = 4).

Model	AUC (95% CI)	∆AUC	LRT *p* (df)	AIC	∆AIC	IDI (95% CI)	cNRI (95% CI)
Base	0.851 (0.833–0.868)	—	—	2075.3	—	—	—
Model A	0.856 (0.837–0.873)	+0.005	<0.001 (1)	2044.5	−30.8	0.0139 (0.0077–0.0199)	0.301 (0.204–0.400)
Model B	0.860 (0.843–0.877)	+0.009	<0.001 (3)	2029.5	−45.8	0.0203 (0.0129–0.0279)	0.255 (0.157–0.354)

### 3.7. Discrimination: Association Versus Prediction

Despite a consistently significant association (Section 3.3), the difference’s standalone discrimination was weak: AUC 0.547 in the full cohort—barely above the 0.5 chance level—rising only to 0.609 in the osteoporosis subgroup and 0.601 in the dual cohort, and falling to 0.522 under the FN anchor (Table 5). These values demonstrate directly that statistical significance in a large sample does not imply predictive utility: the difference on its own cannot reliably classify individuals as fracture or non-fracture. Its informative content is incremental rather than standalone—adding the difference to TH T-score raised AUC from 0.768 (95% CI 0.744–0.789) to 0.785 (0.762–0.808)—a +0.017 increment in this simple two-variable comparison, but with overlapping confidence intervals, so even this increment is not clearly significant—consistent with the modest ΔAUC (+0.005 to +0.009) and IDI (0.014) that is seen when the difference is added to the full covariate model (Table 6). The slightly higher standalone AUC within the osteoporosis range (0.609) does not indicate a stronger relative effect there (the interaction test in Section 3.8 is non-significant) but reflects the higher baseline fracture rate of that population.

**Table 6:** Standalone and combined discrimination (AUC, with 95% bootstrap CIs) of the signed difference across cohorts. The difference-only AUC isolates statistical *association* from individual-level *prediction*; TH T-score alone and TH T-score + difference (full cohort) quantify the incremental contribution.

Model	AUC (95% CI)
Signed difference alone—full cohort	0.547 (0.517–0.576)
Signed difference alone—TH T-score ≤ −2.5	0.609 (0.567–0.651)
Signed difference alone—Dual	0.601 (0.557–0.644)
Signed difference alone—FN anchor	0.522 (0.487–0.557)
Full: TH T-score alone	0.768 (0.744–0.789)
Full: TH T-score + difference	0.785 (0.762–0.808)

### 3.8. Effect-Modification (Interaction) Analysis

None of the interaction terms approached significance: difference × osteoporosis status (TH T-score ≤ −2.5) gave OR 1.08 (95% CI 0.79–1.48, *p* = 0.627 unadjusted; 0.75–1.50, *p* = 0.740 adjusted), and difference × TH T-score as a continuous interaction gave OR 0.94 (0.83–1.07, *p* = 0.368) (Table 7). There is therefore no statistically detectable effect modification; however, interaction tests are underpowered and the adjusted CI (0.75–1.50) cannot exclude modest effect modification in either direction, so we claim only the absence of detectable modification, not strict constancy. Under this reading, the larger absolute fracture rate separation seen in the osteoporosis range (Section 3.2 and Section 4.1) is most parsimoniously attributed to a higher baseline fracture rate rather than to a steeper relative difference effect. We explicitly avoid the common error of inferring effect modification from “significant in subgroup A, non-significant in subgroup B” comparisons; the formal interaction test does not support such a claim.

**Table 7:** Difference × severity interaction (effect modification) terms, testing whether the *relative.* difference effect varies with T-score severity.

Interaction Term	Interaction OR (95% CI)	*p*-Value
difference × osteoporosis (unadjusted)	1.08 (0.79–1.48)	0.627
difference × osteoporosis (adjusted)	1.06 (0.75–1.50)	0.740
difference × TH T-score continuous (adjusted)	0.94 (0.83–1.07)	0.368

### 3.9. Fracture Subtype Analysis

**Figure 7:**
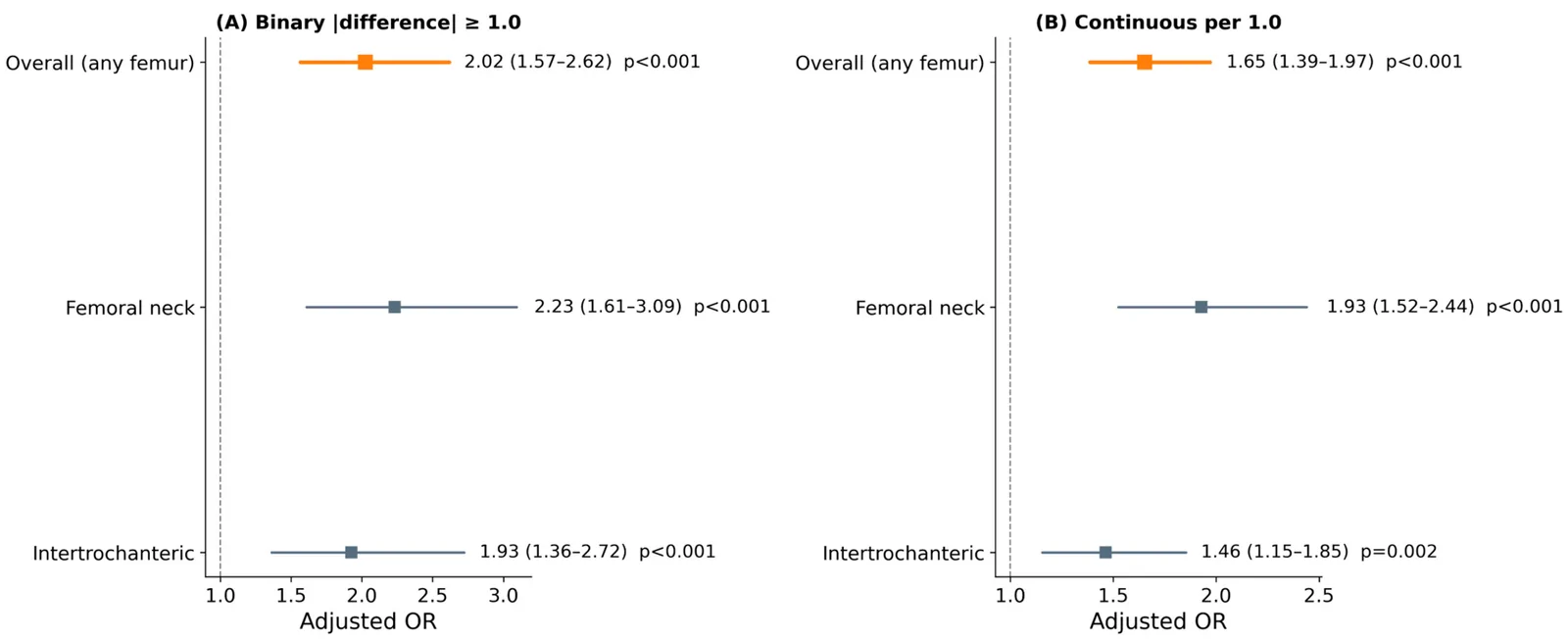
Forest plot of adjusted ORs by fracture subtype (full cohort).

For the femoral neck subtype (219 events, N = 3523), the continuous difference OR was 1.93 (95% CI: 1.52–2.44; *p* < 0.001) and the binary OR 2.23 (95% CI: 1.61–3.09; *p* < 0.001). For the intertrochanteric subtype (229 events, N = 3533), both binary (OR 1.93, 95% CI: 1.36–2.72; *p* < 0.001) and continuous (OR 1.46, 95% CI: 1.16–1.85; *p* = 0.002) analyses were significant (Table 8). The association was directionally consistent across both subtypes (Figure 7). Because the two subtype analyses share the same control pool and are not independent, their effect sizes are not directly compared; only the consistent direction is interpretable.

**Table 8:** Signed-difference associations by fracture subtype (full cohort). Subtype analyses share a common control pool and are not independent; only the consistent direction is interpretable.

Subtype	N	Events	Binary OR (95% CI)	Binary *p*	Continuous OR (95% CI)	Cont *p*
Overall (any femur)	3752	448	2.02 (1.57–2.62)	<0.001	1.65 (1.39–1.97)	<0.001
Femoral neck	3523	219	2.23 (1.61–3.09)	<0.001	1.93 (1.52–2.44)	<0.001
Intertrochanter	3533	229	1.93 (1.36–2.72)	<0.001	1.46 (1.16–1.85)	0.002

### 3.10. Sensitivity and Subgroup Analyses

**Figure 8:**
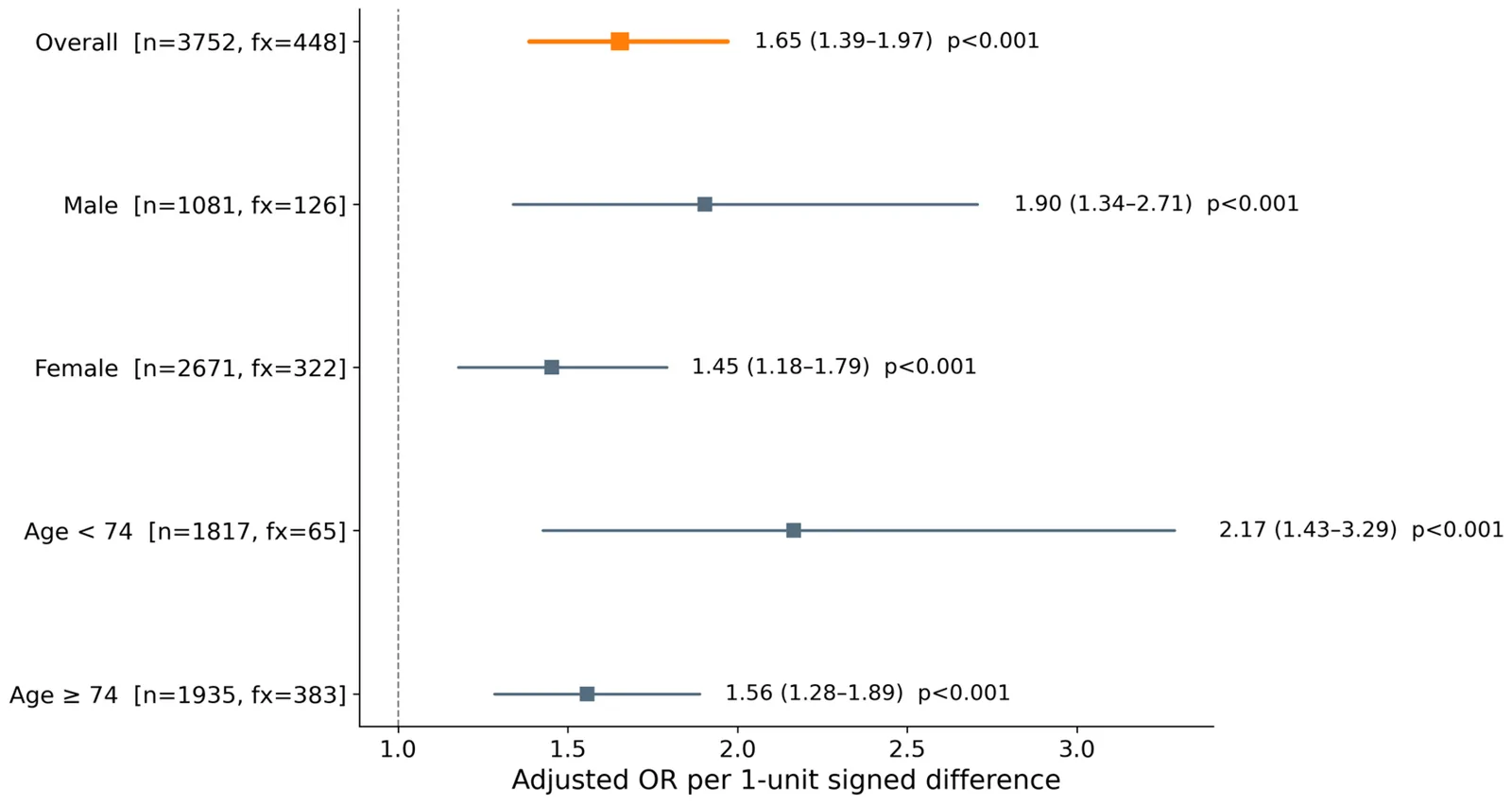
Subgroup forest plot by sex and age (median split at 74 years; full cohort).

The lifestyle-adjusted (OR 1.65, 95% CI: 1.39–1.97) and clinical-only (OR 1.63, 95% CI: 1.37–1.95) models were virtually identical, indicating the association was not confounded by lifestyle variables (Table 9). The effect was significant in both sexes (male OR 1.90, 95% CI: 1.34–2.71; female OR 1.45, 95% CI: 1.18–1.79) and across both age strata, with a numerically larger but less precise estimate among younger patients (age < 74: OR 2.17, 95% CI: 1.43–3.29, 65 events; age ≥ 74: OR 1.56, 95% CI: 1.28–1.89). These subgroup analyses were exploratory and no formal interaction tests were performed; the numerically larger estimate at age <74 should not be over-interpreted given its wide interval and small event count (Figure 8). No between-subgroup difference in effect is claimed; the only formal effect-modification test performed was the difference × severity interaction of Section 3.8, which was non-significant.

**Table 9:** Covariate-set and subgroup sensitivity analyses (continuous signed difference; subgroups exploratory, no formal interaction tests).

Analysis	Group	OR (95% CI)	*p*-Value	N	Events
Covariate set	Lifestyle-adjusted	1.65 (1.39–1.97)	<0.001		
Covariate set	Clinical-only	1.63 (1.37–1.95)	<0.001		
Subgroup	Overall	1.65 (1.39–1.97)	<0.001	3752	448
Subgroup	Male	1.90 (1.34–2.71)	<0.001	1081	126
Subgroup	Female	1.45 (1.18–1.79)	<0.001	2671	322
Subgroup	Age < 74	2.17 (1.43–3.29)	<0.001	1817	65
Subgroup	Age ≥ 74	1.56 (1.28–1.89)	<0.001	1935	383

### 3.11. Osteopenia Range Confirmatory Analysis

**Figure 9:**
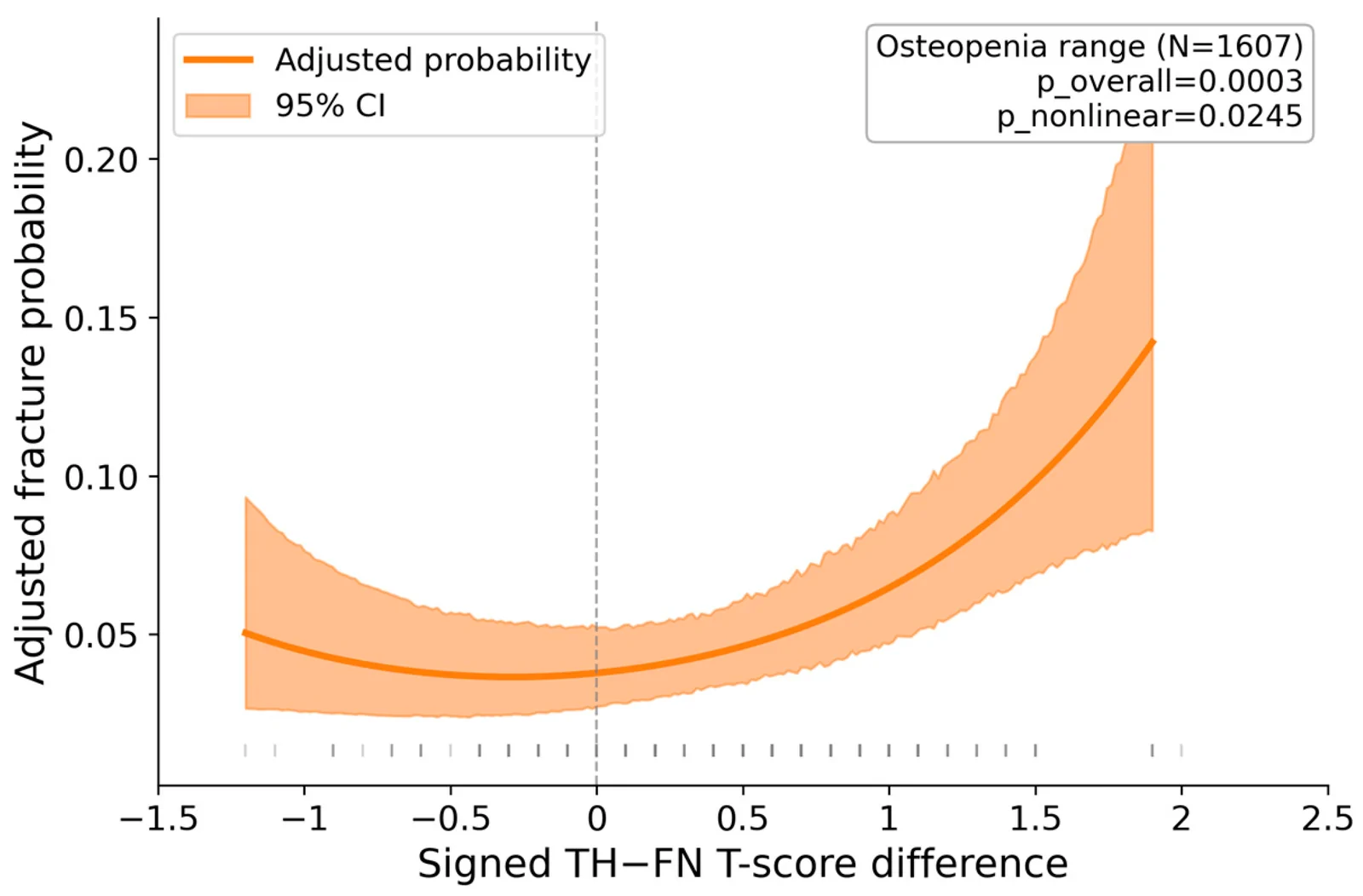
Restricted cubic spline of adjusted fracture probability versus signed TH–FN difference in the osteopenia range (−2.5 < TH T-score ≤ −1.0; CI shading α = 0.5).

In the osteopenia cohort (WHO low-bone-mass range, −2.5 < TH T-score ≤ −1.0 [11]; N = 1607; 149 fractures, 9.3%), the RCS analysis showed a significant overall association (LRT *p*_overall < 0.001) with a nonlinear component (*p*_nonlinear = 0.025). Figure 9 presents the RCS curve. A significant difference signal in the osteopenia range indicates that the association is not confined to the osteoporosis range but operates across the T-score spectrum—concordant with the non-significant interaction (Section 3.8). Given the exploratory nature of this analysis and the cohort’s lower baseline fracture rate (9.3%, which yields smaller absolute separation), it is interpreted as confirmatory support rather than a primary finding.

### 3.12. Inpatient-Restricted Sensitivity Analysis

Because all 448 cases were inpatients whereas controls were a mix of inpatients and outpatients (Section 4.8), the primary models were refitted, restricting controls to inpatients only (cases vs. 1731 inpatient controls; N = 2179) and holding admission status approximately constant. Table 10 shows that the continuous signed-difference association was essentially unchanged from the full cohort (adjusted OR 1.74, 95% CI: 1.44–2.09, vs. 1.65, 1.39–1.97), as was the binary contrast (|difference| ≥ 1.0: OR 2.06, 1.56–2.71), while the standalone discrimination remained weak (difference-alone AUC 0.563). That the relative association persists when admission status is held constant indicates it is not an artifact of the inpatient/outpatient near-separation, although residual confounding by acute-illness severity *within* inpatients cannot be excluded.

### 3.13. Sensitivity to Exposure Scale and Fracture Energy

The per-SD association was unchanged when the exposure was re-expressed on scales independent of the T-score reference databases: OR 1.42 per SD (95% CI 1.26–1.61) for the original T-score difference, 1.43 per SD (1.26–1.62) for the within-cohort z-standardized difference, and 1.47 per SD (1.28–1.68) for the reconstructed areal-BMD difference (correlation with the T-score difference, r = 0.97). Restricting cases to low-energy fractures likewise left the association intact (OR 1.68, 95% CI 1.39–2.02; N = 3699, 395 events). The association is therefore not attributable to reference database scaling or to high-energy trauma cases.

### 3.14. Subtype Case–Case Comparison

Direct comparison among fracture cases (femoral neck, n = 219, versus intertrochanteric n = 229) showed no statistically significant subtype difference in the signed TH–FN difference (crude OR 1.22, 95% CI 0.96–1.55, *p* = 0.103; adjusted OR 1.16, 0.89–1.51, *p* = 0.263). The numerically different subtype-versus-control ORs in Section 3.9 therefore do not constitute evidence of a subtype-specific effect, and we interpret only their consistent direction.

### 3.15. Comparison with the ISCD Lowest-Site Rule

Among the 3696 complete case patients with lumbar T-scores (438 fractures), the lowest of the three site T-scores was the femoral neck in 2286 (61.8%), the lumbar spine in 868 (23.5%), and the total hip in 542 (14.7%), representing the primary association replicated in this subset (OR 1.66, 95% CI 1.39–1.98, TH-adjusted). Decomposing the adjustment clarifies the results (Table 11).

**Table 11:** Signed-difference odds ratios under alternative severity adjustments, including the ISCD low-est-site T-score (N = 3696; 438 fractures).

Severity Adjustment	Signed-Difference OR (95% CI)	*p*
TH T-score (primary specification)	1.66 (1.39–1.98)	<0.001
TH + lumbar T-score	1.69 (1.41–2.02)	<0.001
FN T-score (anchor reversed)	0.87 (0.72–1.05)	0.14
Min (FN, TH)	1.01 (0.85–1.20)	0.91
*T*min = min (lumbar, FN, TH) (ISCD lowest site)	1.04 (0.88–1.23)	0.66 (LRT *p* = 0.66)

Adding the lumbar T-score to the TH adjustment left the association intact, whereas any adjustment containing femoral neck information—including the ISCD lowest-site T-score—abolished it. This is the empirical counterpart of the algebraic identity in Section 2.4: the full-cohort adjusted signal is femoral neck information, which the lowest-site rule already captures. Within the pre-specified TH ≤ −2.5 subgroup, however, the association persisted even after lowest-site adjustment (OR 1.34, 95% CI 1.02–1.76, *p* = 0.034, versus 1.54 under the original specification), indicating that, among patients selected by TH severity, the signed difference retains information beyond the lowest-site T-score; given its magnitude and the absence of multiplicity adjustment, we regard this residual subgroup association as hypothesis-generating. Lumbar T-scores in the elderly can be falsely elevated by degenerative change [30] (maximum +8.1 in this cohort), which would tend to shift the lowest site toward the hip ROIs; this caveat applies to all lowest-site analyses.

## 4. Discussion

### 4.1. Principal Finding

In a full available elderly hip-DXA cohort, the signed TH–FN T-score discordance was *consistently* associated with proximal femur fracture after adjustment for the TH T-score and 12 further clinical covariates. Each 1.0-unit increase in the signed difference—corresponding to a 1 SD relative worsening of FN versus TH—carried a 65% increase in fracture odds (adjusted OR 1.65, 95% CI: 1.39–1.97), and the relative effect showed no statistically detectable variation across severity (difference × osteoporosis interaction OR 1.06, 95% CI 0.75–1.50; difference × TH T-score continuous OR 0.94, 0.83–1.07). The association reproduced across binary, continuous, spline, quintile, incremental value, sensitivity, and subtype analyses; held in both sexes and both age strata; and—addressing the principal design concern—persisted essentially unchanged when controls were restricted to inpatients (adjusted OR 1.74, 95% CI 1.44–2.09; Section 3.12), arguing against the inpatient/outpatient near-separation as its source.

Two findings, however, bound the claim and define the honest headline of this study. First, the difference’s *standalone discrimination is weak*: the difference-only AUC was 0.547 in the full cohort—barely above chance—and only 0.609 in the osteoporosis subgroup (Table 6). A statistically robust association in a large sample (n = 3752) does not translate into the ability to classify individuals; the difference cannot serve as a standalone screening or diagnostic test. Second, the *incremental contribution* of the difference beyond TH severity is real but modest (ΔAUC +0.005 to +0.009 and IDI 0.014 in the full covariate model; a +0.017 AUC increment from 0.768 to 0.785 in the simple TH T-score → TH T-score + difference comparison). Because the signed difference equals TH T-score–FN T-score, this increment is algebraically the value of FN information added on top of TH—a coherent biological quantity, but a supplementary one.

Where, then, is the difference useful? The *relative* effect was comparable in the full cohort and the osteoporosis subgroup (1.65 vs. 1.46), and the interaction test was non-significant, so there is no evidence that the difference acts more strongly in osteoporotic patients. What changes is the absolute fracture rate separation: the unadjusted high- versus low-discordance fracture rate contrast widened from 4.5 percentage points in the full cohort (15.3% vs. 10.8%) to 16.9 points in the osteoporosis subgroup (42.7% vs. 25.8%). This is the arithmetic consequence of applying a near-constant odds ratio to a much higher baseline fracture rate—not effect modification. The practical implication is bounded: the difference is at most a supplementary, hypothesis-generating marker. Any concentration of value in osteoporotic patients would arise through higher absolute fracture rates rather than amplification of the relative effect—but absolute-rate amplification is a generic property of any constant-OR factor applied to a higher baseline and is not specific to this marker, and the absolute rates reported here are within-sample rather than population incidence. We therefore frame the clinical relevance as hypothesis-generating, not established.

The additional analyses sharpen this reading. Re-expressing the exposure on standardized and BMD scales left the association unchanged (Section 3.13), and the lowest-site decomposition (Section 3.15) demonstrated directly that the full-cohort adjusted signal is femoral neck information: it survived additional lumbar adjustment but vanished under any adjustment containing FN, exactly as the algebra of Section 2.4 requires. As to how much of the observed difference reflects biology versus reference systems: a fixed inter-database offset shifts the difference’s location (median ≈ 0.5 SD, Section 2.2) but not its patient-to-patient variation, and the invariance of the association under standardized and BMD-scale re-expression indicates that the modeled gradient reflects patient-level regional heterogeneity rather than reference database scaling—although the absolute location of any single patient’s difference remains a mixture of the two. The residual subgroup association beyond the lowest-site T-score in TH-defined osteoporosis is the only finding not reducible to the lowest-site rule and is presented as hypothesis-generating.

### 4.2. Biological Plausibility

The femoral neck is biomechanically vulnerable, subject to high bending stress during gait and falls; its strength is substantially determined by cortical construction established during growth [31]. Age-related endosteal resorption thins the FN cortex, disproportionately compromising the superolateral cortex, and in the prospective AGES-Reykjavik cohort superior femoral neck cortical thinning discriminated incident hip fracture even after adjustment for femoral neck areal BMD [32]. When FN bone loss outpaces TH loss—producing a positive discordance—it may signal localized acceleration of FN microstructural deterioration not fully captured by the TH composite. Because adjusting for TH T-score and adding the difference is equivalent to adding FN T-score, the difference operationally represents “FN beyond TH,” which offers a plausible mechanism for the small but reproducible incremental signal—consistent with the long-established primacy of femoral neck BMD as the single best densitometric predictor of hip fracture [26]. The subtype results are consistent with an FN cortex mechanism—the femoral neck subtype showed a numerically larger continuous difference OR (1.93) than the intertrochanteric subtype (1.46)—but because the two subtype analyses share the same control pool and are not independent, these magnitudes are not directly compared, and only the consistent direction is interpretable. We caution that the intertrochanteric subtype association is not obviously explained by an FN cortex mechanism and more likely reflects global proximal femur fragility correlated with FN deterioration; the FN cortex account is specific to the femoral neck subtype. These subtype-specific differences are consistent with a broader literature showing that regional proximal femur BMD differs between femoral neck and intertrochanteric fractures [18,19,20,21,33]. More broadly, a patient whose femoral neck is disproportionately worse than the total hip may harbor localized cortical fragility that global BMD summaries average away. Because the signed TH–FN difference captures this regional skeletal heterogeneity not fully represented by the total hip T-score alone, it is best positioned as an *adjunctive imaging biomarker* that complements established fracture risk tools such as FRAX—or future machine-learning models trained on DXA—rather than replacing them.

### 4.3. Comparison with Prior Discordance Literature

Prior work has primarily characterized the prevalence and determinants of inter-site and intra-hip discordance [14,17], with less attention to its directional relationship with fracture. Two lines of prior work are closest to ours: hip–spine BMD discordance has been associated with hip fracture in Korean elderly (odds ratios ≈ 2.9–3.4) [34], although that contrast is inter-site (hip versus spine) rather than intra-hip; and a localized femoral neck-minus-trochanter T-score difference has been compared across hip-fracture sites [23], echoing an early observation that a signed inter-site BMD contrast independently discriminates fracture type [22]. Those reports, however, compared fracture types among fracture patients (without fracture-free controls) and did not quantify standalone discrimination. Our results extend that literature in a specific and rigorous way: working in a full available cohort with fracture-free controls, we show that the directional TH–FN difference is robustly associated with fracture, yet—unlike the framing sometimes attached to discordance—is not a strong individual-level predictor. The contribution is therefore methodological as much as substantive: to our knowledge this is the first study to quantify, for a routinely derivable intra-hip discordance measure, the gap between a genuine spectrum-wide association and weak individual-level discrimination—by reporting standalone AUC and a formal interaction test alongside ORs, we make explicit a distinction that is often blurred when only *p*-values or odds ratios are reported in large samples. We regard this association–prediction separation, rather than any new diagnostic marker, as the principal value of the study.

### 4.4. Total Hip as the Preferred Anchor and the FN Anchor Symmetry

Appendix A shows that under an FN anchor (FN T-score ≤ −2.5; N = 1378), the continuous difference remained associated with fracture (OR 1.92, 95% CI: 1.47–2.51; *p* < 0.001) while the binary contrast was weaker (OR 1.57, 95% CI: 1.12–2.19; *p* = 0.008) and the RCS nonlinearity was absent (*p*_nonlinear = 0.692). The symmetric FN anchor series (Table A2) confirms that FN-referenced differences against all other ROIs (FN–TH, FN–TR, FN–IT) are associated with fracture, but all with similarly weak standalone discrimination (difference-alone AUC 0.522–0.564). Anchoring the pre-specified subgroup on TH is *not* a claim that TH is the densitometric reference site—on the contrary, the WHO/NHANES young-adult reference and FRAX both privilege the femoral neck. The TH anchor is preferred here on pragmatic grounds: TH has lower in-vivo precision error and better reproducibility than FN, and the difference behaved more consistently under a TH anchor. Critically, the primary full-cohort analysis requires no anchoring at all, since it adjusts for TH T-score directly.

### 4.5. The Osteopenia Signal

A significant difference–fracture association was present in the osteopenia range (*p*_overall < 0.001), which is exactly what the spectrum-wide, no-interaction interpretation predicts: if the relative effect is constant across severity, a signal should persist below the osteoporosis threshold. The osteopenia analysis therefore functions as confirmatory support, not as an independent primary finding. Two caveats apply: the lower baseline rate (9.3%) yields smaller absolute separation, and the control population mixes inpatients and outpatients (Section 4.8).

### 4.6. Clinical Implications

If externally validated, the signed TH–FN difference could be reported alongside routine hip DXA at no additional cost—it requires only arithmetic on already-measured values. In practical terms, of two patients with an identical total hip T-score, the one with the relatively lower femoral neck (a positive TH–FN difference) carries modestly higher fracture odds; although far too weak to stand alone, this cost-free, already-measured quantity may help clinicians recognize regional skeletal heterogeneity when interpreting routine DXA. Its appropriate role, however, is narrow and supplementary. It is not a screening test (standalone AUC ≈ 0.55), not a diagnostic or treatment threshold (the |difference| ≥ 1.0 contrast is interpretive, not validated as a cutoff), and not a marker whose effect is amplified in osteoporosis (interaction non-significant). Where the baseline fracture rate is high, the same relative association does correspond to a larger absolute fracture rate difference [35], but (as above) this is a generic property of constant-OR markers and the rates here are within-sample. The marker is also largely redundant with tools already in routine use: ISCD directs diagnosis to the *lowest* site T-score, so a worse FN is already operative [12], and FRAX takes femoral neck BMD as its skeletal input [36]—precisely the “FN-beyond-TH” quantity isolated here. Any incremental role would therefore have to be demonstrated *over* FRAX and the lowest-T-score rule, which we could not test. Consistent with this redundancy, the lowest-site decomposition (Section 3.15) showed no information beyond the ISCD lowest-site T-score in the full cohort—our data thus provide direct quantitative support for the lowest-site practice itself. The only potential niche is the residual, hypothesis-generating association observed within TH-defined osteoporosis (OR 1.34 beyond the lowest-site T-score), which—if prospectively confirmed with decision-analytic evaluation, ideally integrated with FRAX—might inform attention to FN-dominant loss. We advance no treatment-selection claim on the present data.

### 4.7. Strengths

1. **Full-cohort primary design (N = 3752).** Using the fully available complete-case cohort and adjusting for TH T-score directly maximizes power, avoids selection on the exposure-defining variable, and forestalls cherry-picking concerns; the pre-specified osteoporosis subgroup deepens rather than defines the primary analysis.
2. **Explicit association-versus-prediction reporting.** By reporting standalone AUC and a formal interaction test alongside ORs, we avoid over-interpreting statistical significance in a large sample as predictive or effect-modifying utility.
3. **Multiple converging analytic approaches.** Binary, continuous, spline, quintile, incremental-value, sensitivity, and subtype analyses reached concordant conclusions, ruling out threshold dependence.
4. **Rigorous data curation.** A documented audit recovered nine height–weight transpositions, corrected leading-digit and typographical errors, recalculated BMI for every row, and rebuilt the diabetes covariate from free text.
5. **Reproducibility controls.** A fixed seed and function-local bootstrap generators (2000 resamples) make interval estimates order-independent and reproducible.
6. **Robustness to admission-status confounding.** The primary association persisted essentially unchanged when controls were restricted to inpatients (Section 3.12; adjusted OR 1.74, 95% CI 1.44–2.09), and the incremental signal was confirmed out-of-sample by cross-validation with no optimism inflation (calibration slope 1.00; VIF 1.05), strengthening internal validity.

### 4.8. Limitations

1. **Weak standalone discrimination; hypothesis-generating only.** The difference-alone AUC (0.547 full; 0.609 osteoporosis) shows that the marker cannot classify individuals on its own. All findings are hypothesis-generating and require prospective validation; the marker is supplementary, not a stand-alone tool.
2. **Inpatient/outpatient near-separation.** All fracture cases were inpatients by design, whereas controls were 52.4% inpatient (SMD = 1.348). Because admission status is almost perfectly aligned with case status, source type cannot be adjusted for and may confound comparisons; absolute fracture rates here should not be read as community incidence.
3. **Retrospective single-center case–control design.** Results may not generalize to other ethnic groups, DXA devices, or care settings; in particular, the magnitude and distribution of TH–FN discordance—and potentially its association with fracture—may differ across populations with different skeletal geometry, body composition, patterns of age-related bone loss, and normative reference databases, so external validation in other populations and DXA systems is required. Additionally, unmatched controls may introduce selection bias from unmeasured confounders (e.g., fall risk, frailty) and, because controls underwent DXA for clinical indications (Section 2.1), they are not a random sample of the source population, but a further potential source of selection bias. Conversely, for an intra-hip *difference* measure, the single-center design is also a strength: because absolute BMD and derived T-scores differ systematically between DXA manufacturers and even between state-of-the-art scanners [37], acquiring every scan on one device under uniform settings minimizes between-instrument variance in the TH–FN contrast and improves the internal consistency of the exposure.
4. **Text-parsed comorbidity flags.** Hypertension, stroke, and lipid-disorder flags were derived from free-text fields and were undercounted by the parser; they were therefore excluded from the covariate set, leaving potential residual confounding; more broadly, clinically relevant variables not captured in this retrospective dataset—fall propensity, frailty, functional status, and medication exposures beyond osteoporosis treatment—could not be adjusted for, so residual confounding from unavailable or incompletely captured variables cannot be excluded.
5. **Concurrent (peri-fracture) DXA timing.** In some cases the qualifying DXA was obtained within 2 weeks of the index fracture. This is before surgery and on the contralateral hip, which avoids direct fracture-site artefact, although acute pain, positioning limitations, immobilization, or other peri-fracture factors could still subtly affect measurement. The exposure is therefore measured essentially concurrently with the outcome, so the design is cross-sectional in timing and supports an association rather than prospective prediction, and time-to-event analysis was not applicable.
6. **Absence of FRAX and lumbar spine.** FRAX variables were not available in this retrospective dataset—cases were ascertained largely through emergency presentation with acute fracture requiring surgery, so the outpatient inputs FRAX requires were not systematically recorded—so we could not benchmark the difference’s incremental value against FRAX. Prompted by peer review, we re-audited the source data and found that lumbar spine T-scores were in fact extractable from the structured DXA reports for 98.5% of patients; the lowest-site analyses of Section 3.15 were added on this basis. Lumbar T-scores in the elderly remain susceptible to false elevation from degenerative change [30], a caveat noted in Section 3.15.
7. **Effect-modification not claimed; subtypes not compared.** The interaction test was non-significant, so we do not claim the difference acts more strongly in osteoporosis; and because the two subtypes share a control pool, their effect sizes are not directly compared.
8. **Exploratory analyses do not establish utility.** The TR/IT ROIs, ROI pair, and machine-learning analyses (Appendix B and Appendix C) are exploratory; cross-validated machine-learning AUC gains do not establish clinical utility.
9. **Absolute fracture proportions are within-sample, not incidence.** Under the case–control sampling and the inpatient/outpatient near-separation, the reported fracture rates and percentage-point contrasts are not population incidence; translation into absolute risk estimates requires a cohort with defined follow-up.
10. **Conceptual overlap with existing densitometric practice.** The adjusted signal is algebraically femoral neck information beyond total hip—the quantity underlying the ISCD lowest-site rule and FRAX’s FN input; the contribution is the rigorous association-versus-discrimination characterization, not a new biological marker.
11. **Concurrent timing; multiplicity; potential harms.** Because the qualifying DXA in cases was obtained within 2 weeks of the fracture, the exposure is measured essentially concurrently with the outcome; the relationship is therefore cross-sectional in timing rather than demonstrably antecedent, and prospective validation is required to establish true temporal precedence. The many secondary cohorts, ROI pairs, subtypes, and subgroups carry unadjusted, hypothesis-generating *p*-values. Additionally, as a weak standalone flag (AUC ≈ 0.55) the marker could cause false reassurance—a negative difference must never down-weight an existing osteoporosis treatment indication—or over-testing.

### 4.9. Future Directions

Prospective, multi-center validation with structured fracture dates would permit time-to-event analysis and direct benchmarking against FRAX. Restriction to inpatient controls would remove the admission-status near-separation. Incorporating lumbar spine BMD, and testing whether a positive discordance flag improves decision-making among osteoporotic patients in a prospective design, are the natural next steps before any clinical adoption.

## 5. Conclusions

In a full available elderly hip-DXA cohort, the signed TH–FN T-score discordance was consistently associated with proximal femur fracture after adjustment for the TH T-score and 12 further covariates (adjusted OR 1.65 per 1.0 unit; 95% CI: 1.39–1.97), with no statistically detectable variation across severity and persistence when controls were restricted to inpatients (OR 1.74). Its *standalone discrimination*, however, was weak (AUC 0.547 full; 0.609 osteoporosis) and its incremental value over TH severity was negligibly out-of-sample (cross-validated ΔAUC +0.005, CI including zero), meaning that it is not a standalone predictor and not a diagnostic or treatment threshold. Because the adjusted signal is algebraically femoral neck information beyond total hip, it overlaps substantially with the ISCD lowest-site rule and FRAX’s femoral neck input—an overlap we now demonstrate directly: adjusting for the ISCD lowest-site T-score (including the lumbar spine) abolished the full-cohort association, while a residual, hypothesis-generating association persisted within TH-defined osteoporosis (Section 3.15); any larger absolute fracture rate separation in osteoporotic patients is a generic consequence of a higher baseline fracture rate (and within-sample, not incidence), not effect modification. The contribution of this work is therefore twofold: it provides a quantitative, multi-method characterization of a routinely derivable intra-hip discordance measure—available from standard hip DXA at no additional cost—and it offers a clear demonstration that a robust large-sample association need not confer individual-level discriminative utility. On this basis the signed difference is best regarded as a supplementary, hypothesis-generating marker; prospective validation, benchmarking over FRAX, and absolute-risk estimation in a follow-up cohort are the necessary next steps before any clinical application. Patients with marked positive TH–FN discordance in TH-defined osteoporosis may merit closer skeletal assessment despite similar TH T-score severity—a possibility that remains to be confirmed prospectively before this readily available DXA-derived measure could complement, never replace, established tools such as FRAX and the ISCD lowest-site rule.

## Figures and Tables

**Table 1:** Study cohorts: definitions, complete case sample size (N), fracture counts, and analytic role.

Cohort	Definition	N (CC)	Fractures	Role
Full cohort	Complete case	3752	448 (11.9%)	**Primary analysis**
TH T-score ≤ −2.5 subgroup	Osteoporosis range	913	262 (28.7%)	Pre-specified subgroup
Dual sensitivity	TH T-score and FN T-score both ≤ −2.5	819	246 (30.0%)	Sensitivity
Osteopenia range	−2.5 < TH T-score ≤ −1.0	1607	149 (9.3%)	Confirmatory (exploratory)
FN anchor	FN T-score ≤ −2.5	1378	333 (24.2%)	Appendix A

**Table 10:** Inpatient-restricted sensitivity analysis (controls restricted to inpatients; cases vs. 1731 inpatient controls; N = 2179, 448 fractures).

Model	OR (95% CI)	*p*-Value
Signed difference, continuous, unadjusted	1.32 (1.14–1.53)	<0.001
Signed difference, continuous, adjusted	1.74 (1.44–2.09)	<0.001
|difference| ≥ 1.0, adjusted	2.06 (1.56–2.71)	<0.001

**Figure A1:**
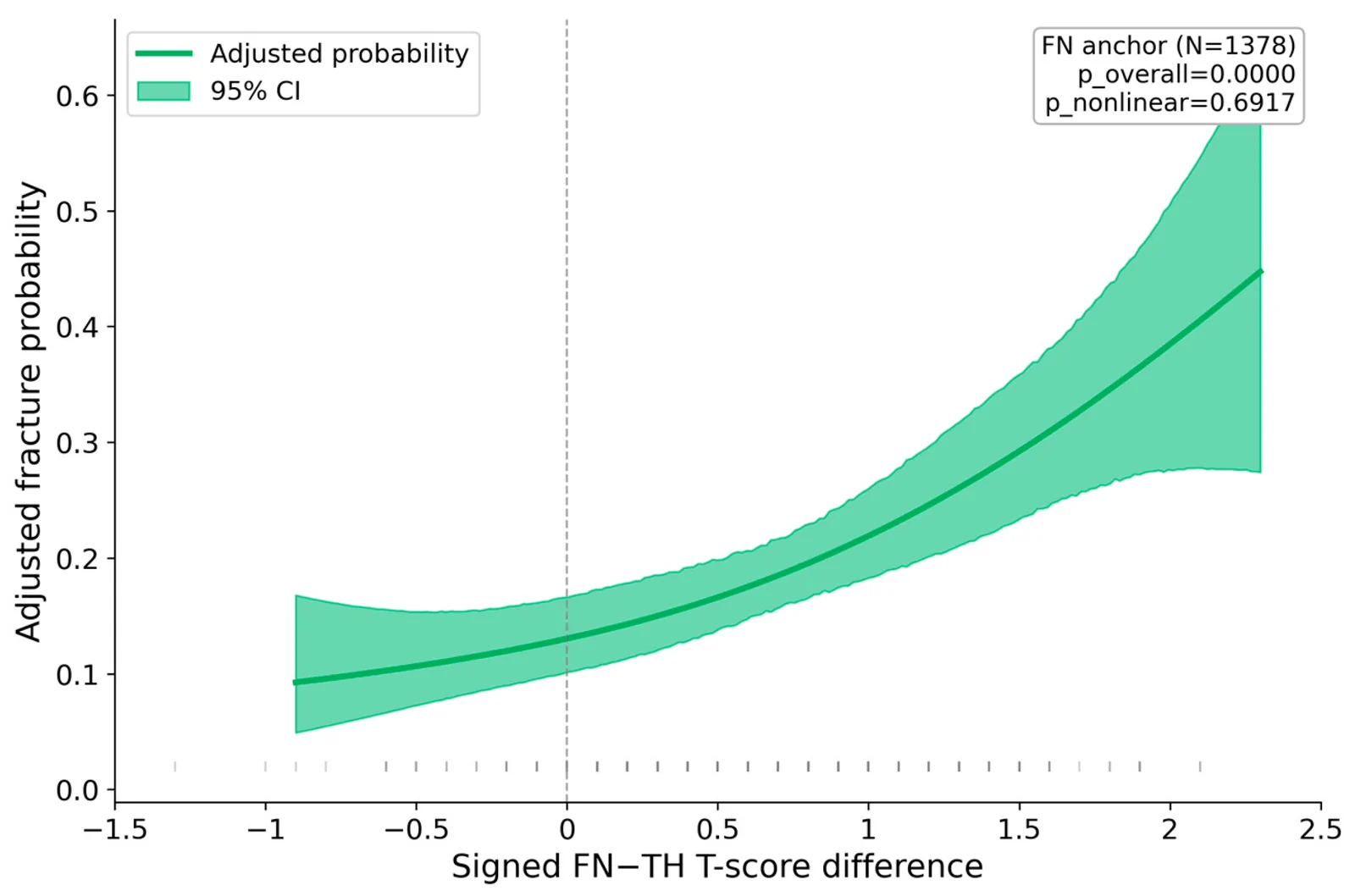
Restricted cubic spline (RCS) of adjusted femur fracture probability versus the signed FN–TH difference, computed within the FN anchored cohort (patients with FN T-score ≤ −2.5; N = 1378, 333 fractures). The “FN anchor” mirrors the total-hip anchor of the main analysis: instead of restricting on TH severity, the cohort is restricted to FN-defined osteoporosis, and the difference is referenced to FN. The curve is the model-predicted probability (linear beyond the outer knots); shading is the 95% CI (α = 0.5). *p*_overall < 0.001, *p*_nonlinear = 0.692 (no nonlinear component).

**Figure A2:**
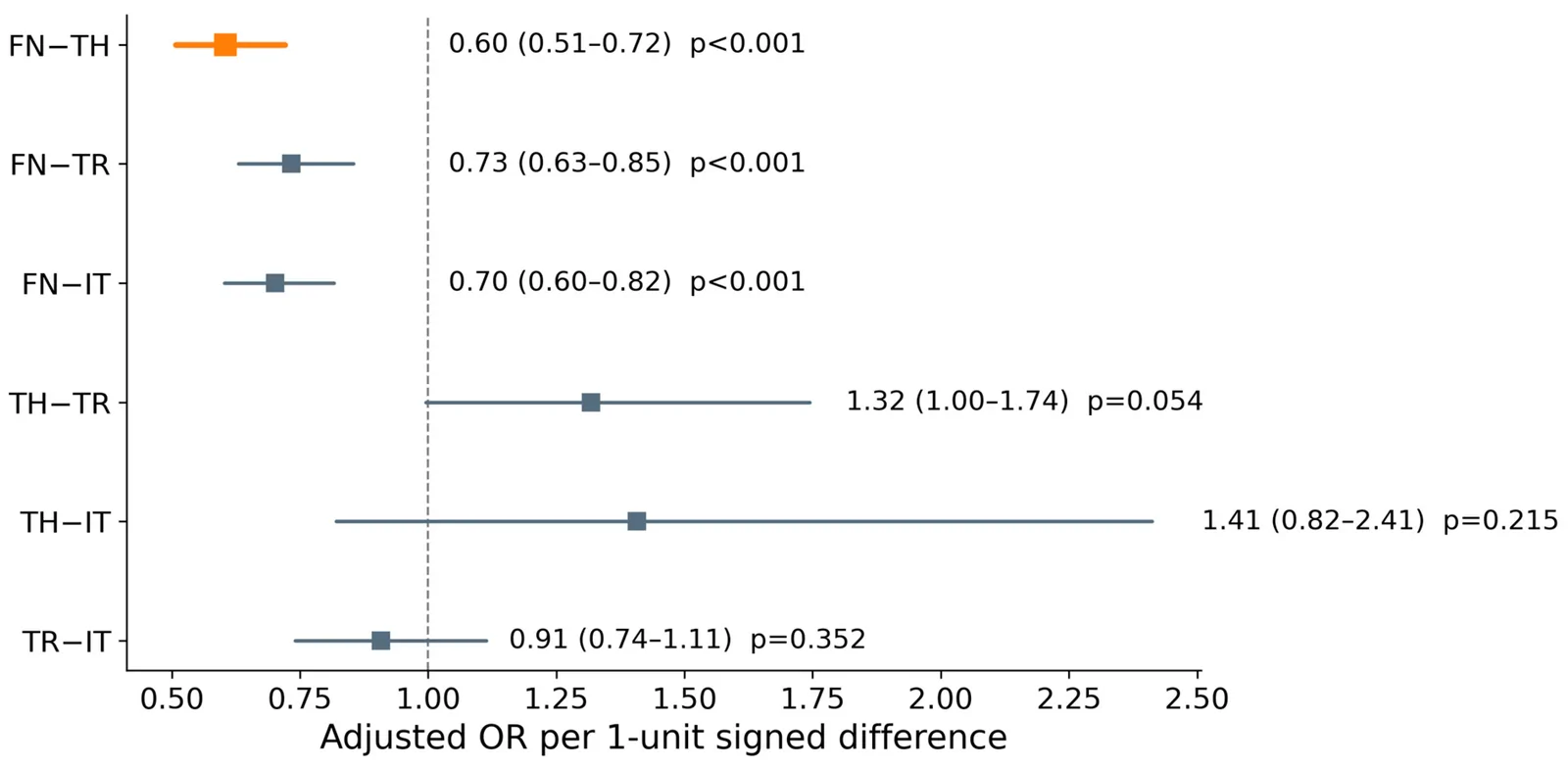
Forest plot of the six unique pairwise ROI signed-difference analyses in the full cohort (N = 3752). Each row (*y*-axis) is one signed difference between two of the four hip ROIs (FN–TH, FN–TR, FN–IT, TH–TR, TH–IT, TR–IT); the *x*-axis is the adjusted odds ratio per 1.0-unit increase in that difference (square = point estimate, whisker = 95% CI, vertical dashed line = OR 1.0, no effect). The principal exposure (TH–FN, shown here in its reverse form, FN–TH) is highlighted. Only FN- involving pairs reach significance, indicating the femoral neck drives the discordance signal.

**Figure A3:**
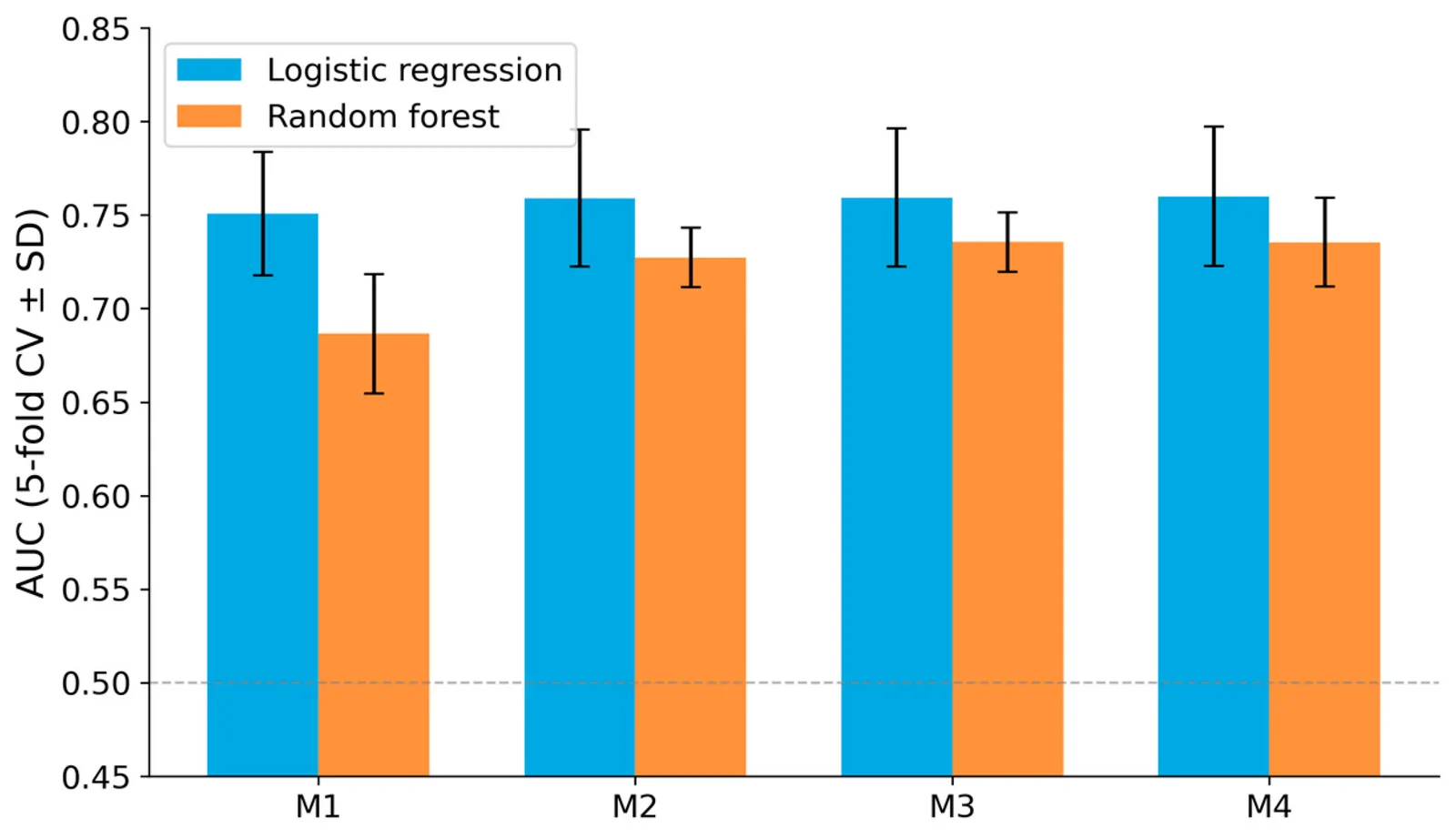
Cross-validated AUC by feature set (LR and RF; error bars = SD; TH T-score ≤ −2.5 subgroup).

## Data Availability

The data presented in this study are not publicly available due to privacy and ethical restrictions. However, data may be available upon reasonable request to the corresponding author, as this study has been exempted from review by the Institutional Review Board (IRB) of Gyeongsang National University Changwon Hospital (GNUCH).

## Appendix A. Total Hip vs. Femoral Neck Anchor Comparison

To assess the transparency of the total-hip anchor used for the pre-specified subgroup, we repeated the binary and continuous difference analyses in the FN anchored cohort (FN T-score ≤ −2.5; N = 1378; 333 fractures, 24.2%), and—symmetric to the TH-anchor ROI exploration—we ran the full series of FN-referenced differences (FN–TH, FN–TR, FN–IT) through binary, continuous, RCS, and standalone-AUC analyses.

**Table A1:** Binary and continuous difference analysis—FN anchor cohort (FN T-score ≤ −2.5; N = 1378, Fx = 333).

Analysis	N	Events	OR (95% CI)	*p*-Value
Binary |difference| ≥ 1.0	1378	333	1.57 (1.12–2.19)	0.008
Continuous per 1.0	1378	333	1.92 (1.47–2.51)	<0.001

**Table A2:** FN anchor series—FN-referenced signed differences against each other ROI (FN T-score ≤ −2.5; N = 1378, Fx = 333). Continuous OR is per 1.0-unit increase in the FN-X difference.

ROI Pair (FN-X)	Binary OR (95% CI)	Bin *p*	Continuous OR (95% CI)	Cont *p*	Difference-Alone AUC	RCS *p*_Overall
FN–TH *	1.57 (1.12–2.19)	0.008	0.52 (0.40–0.68)	<0.001	0.522	<0.001
FN–TR	1.55 (1.15–2.09)	0.004	0.70 (0.57–0.87)	0.002	0.564	0.008
FN–IT	1.36 (1.03–1.81)	0.032	0.62 (0.49–0.78)	< 0.001	0.547	<0.001

* FN–TH is the principal exposure expressed in the reverse direction (FN–TH = −(TH–FN)); its continuous OR of 0.52 is the reciprocal of the corresponding FN anchor TH–FN estimate (1.92).

The continuous difference remained associated with fracture under the FN anchor (OR 1.92; *p* < 0.001), while the binary contrast was weaker (OR 1.57; *p* = 0.008). RCS analysis in this cohort showed a significant overall association (*p*_overall < 0.001) with no nonlinear component (*p*_nonlinear = 0.692). The symmetric series (Table A2) shows that FN-referenced differences against all three other ROIs are associated with fracture but with uniformly weak standalone discrimination (difference-alone AUC 0.522–0.564), mirroring the TH anchor findings. These results confirm that the difference–fracture association does not depend on the choice of anchor and that the primary full-cohort analysis—which requires no anchor at all—captures the effect across the full severity range.

Figure A1 RCS curve for the FN anchored cohort (CI shading at α = 0.5).

## Appendix B. ROI-Pair Exploration

We explored all six unique signed difference pairs derivable from the four hip ROIs in the full complete-case cohort (N = 3752), using the full-cohort adjusted model.

**Table A3:** Adjusted ORs per 1-unit signed difference—six unique ROI pairs (full cohort, N = 3752).

ROI Pair	OR per 1-Unit (95% CI)	*p*-Value
FN–TH *	0.60 (0.51–0.72)	<0.001
FN–TR	0.73 (0.63–0.85)	<0.001
FN–IT	0.70 (0.60–0.82)	<0.001
TH–TR	1.32 (1.00–1.74)	0.054
TH–IT	1.41 (0.82–2.41)	0.21
TR–IT	0.91 (0.74–1.11)	0.35

* FN–TH is the principal exposure expressed in the reverse direction: FN–TH = −(TH–FN). Its OR of 0.60 is the reciprocal of the primary TH–FN estimate (≈1.65), so a 1-unit increase in FN–TH (FN relatively better than total) corresponds to lower fracture odds. The FN–TH pair is the only one whose effect is robust and large; pairs not involving the FN (TH–TR, TH–IT, TR–IT) were not significant, consistent with the femoral neck driving the discordance signal.

Figure A2 forest plot of adjusted ORs for all six ROI-pair signed difference analyses, with the principal TH–FN (=FN–TH) pair highlighted.

## Appendix C. Machine Learning Exploratory Analysis

These analyses are exploratory and remain secondary to the prespecified multivariable analyses; they establish neither incremental clinical utility nor a validated prediction model.

To contextualize the incremental value of the signed difference from a machine-learning perspective, we compared four feature sets using logistic regression (LR) and random forest (RF) classifiers with 5-fold stratified cross-validation in the TH T-score ≤ −2.5 subgroup (N = 913). This analysis is exploratory and reported only here.

**Table A4:** AUC across four feature-set models (5-fold cross-validation; TH T-score ≤ −2.5 subgroup, N = 913).

Model	LR AUC (Mean ± SD)	RF AUC (Mean ± SD)	Top-3 Features (RF)
M1: Clinical only	0.751 ± 0.033	0.687 ± 0.032	BMI, Age_years, Dementia
M2: + 4 T-scores	0.759 ± 0.037	0.727 ± 0.016	Age_years, BMI, FN T-score
M3: + Signed TH–FN	0.759 ± 0.037	0.736 ± 0.016	Age_years, BMI, FN T-score
M4: + All 6 ROI differences	0.760 ± 0.037	0.735 ± 0.024	Age_years, BMI, FN T-score

Adding the signed difference (M3 vs. M2) left cross-validated LR AUC essentially unchanged (both 0.759) and produced a small RF AUC increase (0.736 vs. 0.727). This contrasts with the significant LRT improvement in the logistic incremental analysis (Table 5) and reflects the difference between cross-validated out-of-sample discrimination (which penalizes added complexity) and in-sample LRT (which tests coefficient significance). The difference carries a statistically independent in-sample signal, but this does not by itself establish clinical utility—consistent with the modest ΔAUC (Table 5) and the weak standalone AUC (Table 6). Age and BMI consistently ranked among the top RF predictors across all feature sets.

Figure A3 bar chart of AUC by feature set (LR and RF, with SD error bars; CI/error shading at α = 0.5).

## Appendix D. Quintile Sensitivity Analysis

Quintiles were formed by deterministic first-rank assignment in the full cohort, reporting the difference range, n, fractures, and unadjusted rate per quintile.

**Table A5:** Equal-N quintile distribution of the signed TH–FN difference and unadjusted fracture rate (full cohort).

Quintile	Difference Range	n	Fractures	Rate (%)
Q1 (lowest)	−4.40 to −0.10	750	80	10.7
Q2	−0.10 to 0.30	750	77	10.3
Q3	0.30 to 0.60	751	85	11.3
Q4	0.60 to 1.00	750	98	13.1
Q5 (highest)	1.00 to 3.50	751	108	14.4

Unadjusted fracture rates rose from 10.7% in Q1 to 14.4% in Q5, with the increase concentrated in the upper two quintiles (Q4 13.1%, Q5 14.4%), consistent with the positive-difference steepening seen in the spline. The modest absolute spread across quintiles (10.3–14.4%) again illustrates that, although the gradient is directionally consistent, the difference by itself separates fracture rates over only a narrow absolute range. Figure A4 presents this gradient as a bar chart.

## References

[1] Ensrud K.E., Crandall C.J. (2017). Osteoporosis. Ann. Intern. Med..

[2] Cooper C., Campion G., Melton L.J. (1992). Hip fractures in the elderly: A world-wide projection. Osteoporos. Int..

[3] Yoon H.-K., Park C., Jang S., Jang S., Lee Y.-K., Ha Y.-C. (2011). Incidence and mortality following hip fracture in Korea. J. Korean Med. Sci..

[4] Ha Y.-C., Park Y.-G., Nam K.W., Kim S.-R. (2015). Trend in hip fracture incidence and mortality in Korea: A prospective cohort study from 2002 to 2011. J. Korean Med. Sci..

[5] Cummings S.R., Melton L.J. (2002). Epidemiology and outcomes of osteoporotic fractures. Lancet.

[6] Parker M., Johansen A. (2006). Hip fracture. BMJ..

[7] Johnell O., Kanis J.A. (2006). An estimate of the worldwide prevalence and disability associated with osteoporotic fractures. Osteoporos. Int..

[8] Kanis J.A. (2002). Diagnosis of osteoporosis and assessment of fracture risk. Lancet.

[9] Blake G.M., Fogelman I. (2007). The role of DXA bone density scans in the diagnosis and treatment of osteoporosis. Postgrad. Med. J..

[10] Siris E.S., Brenneman S.K., Miller P.D., Barrett-Connor E., Chen Y.-T., Sherwood L.M., A Abbott T. (2004). Predictive value of low BMD for 1-year fracture outcomes is similar for postmenopausal women ages 50–64 and 65 and older: Results from the National Osteoporosis Risk Assessment (NORA). J. Bone Miner. Res..

[11] World Health Organization (1994). Assessment of Fracture Risk and Its Application to Screening for Postmenopausal Osteoporosis.

[12] Binkley N., Bilezikian J.P., Kendler D.L., Leib E.S., Lewiecki E.M., Petak S.M. (2006). International Society for Clinical Densitometry. Official Positions of the International Society for Clinical Densitometry and Executive Summary of the 2005 Position Development Conference. J. Clin. Densitom..

[13] Lewiecki E.M., Gordon C.M., Baim S., Leonard M.B., Bishop N.J., Bianchi M.-L., Kalkwarf H.J., Langman C.B., Plotkin H., Rauch F. (2008). International Society for Clinical Densitometry 2007 Adult and Pediatric Official Positions. Bone.

[14] Woodson G. (2000). Dual X-ray absorptiometry T-score concordance and discordance between the hip and spine measurement sites. J. Clin. Densitom..

[15] Lu Y., Genant H.K., Shepherd J., Zhao S., Mathur A., Fuerst T.P., Cummings S.R. (2001). Classification of osteoporosis based on bone mineral densities. J. Bone Miner. Res..

[16] Mounach A., Abayi D.M., Ghazi M., Ghozlani I., Nouijai A., Achemlal L., Bezza A., El Maghraoui A. (2009). Discordance between hip and spine bone mineral density measurement using DXA: Prevalence and risk factors. Semin. Arthritis Rheum..

[17] El Maghraoui A., Roux C. (2008). DXA scanning in clinical practice. QJM.

[18] Li N., Yuan Y., Yin L., Yang M., Liu Y., Zhang W., Ma K., Zhou F., Cheng Z., Wang L. (2023). Site-specific differences in bone mineral density of the proximal femur correlate with the type of hip fracture. Diagnostics.

[19] Kanazawa T., Ohmori T., Toda K., Ito Y. (2023). Relationship between site-specific bone mineral density in the proximal femur and instability of proximal femoral fractures: A retrospective study. Orthop. Traumatol. Surg. Res..

[20] Gnudi S., Ripamonti C., Lisi L., Fini M., Giardino R., Giavaresi G. (2002). Proximal femur geometry to detect and distinguish femoral neck fractures from trochanteric fractures in postmenopausal women. Osteoporos. Int..

[21] Cho Y., Lee I., Ha S.H., Park J.H., Park J.H. (2020). Comparison of hip subregion bone mineral density to the type of proximal femur fracture. Arch. Osteoporos..

[22] Greenspan S.L., Myers E.R., Maitland L.A., Kido T.H., Krasnow M.B., Hayes W.C. (1994). Trochanteric bone mineral density is associated with type of hip fracture in the elderly. J. Bone Miner. Res..

[23] Hong T.H., Moon K.H. (2015). Localized femoral BMD T-scores according to the fracture site of hip and the evaluation of the sensitivity of the high risk group designated by FRAX^®^ in hip fracture patients. Osteoporos. Sarcopenia..

[24] Von Elm E., Altman D.G., Egger M., Pocock S.J., Gøtzsche P.C., Vandenbroucke J.P., Initiative S. (2007). STROBE Initiative. The Strengthening the Reporting of Observational Studies in Epidemiology (STROBE) statement: Guidelines for reporting observational studies. Lancet.

[25] Looker A.C., Wahner H.W., Dunn W.L., Calvo M.S., Harris T.B., Heyse S.P., Johnston C.C., Lindsay R. (1998). Updated data on proximal femur bone mineral levels of US adults. Osteoporos. Int..

[26] Marshall D., Johnell O., Wedel H. (1996). Meta-analysis of how well measures of bone mineral density predict occurrence of osteoporotic fractures. BMJ.

[27] Harrell F.E. (2015). Regression Modeling Strategies: With Applications to Linear Models, Logistic and Ordinal Regression, and Survival Analysis.

[28] Pencina M.J., D’Agostino R.B., D’Agostino R.B., Vasan R.S. (2008). Evaluating the added predictive ability of a new marker: From area under the ROC curve to reclassification and beyond. Stat. Med..

[29] Steyerberg E.W., Vickers A.J., Cook N.R., Gerds T., Gonen M., Obuchowski N., Pencina M.J., Kattan M.W. (2010). Assessing the performance of prediction models: A framework for traditional and novel measures. Epidemiology.

[30] Liu G., Peacock M., Eilam O., Dorulla G., Braunstein E., Johnston C.C. (1997). Effect of osteoarthritis in the lumbar spine and hip on bone mineral density and diagnosis of osteoporosis in elderly men and women. Osteoporos. Int..

[31] Zebaze R.M.D., Jones A., Knackstedt M., Maalouf G., Seeman E. (2007). Construction of the femoral neck during growth determines its strength in old age. J. Bone Miner. Res..

[32] Johannesdottir F., Poole K.E., Reeve J., Siggeirsdottir K., Aspelund T., Mogensen B., Jonsson B.Y., Sigurdsson S., Harris T.B., Gudnason V.G. (2011). Distribution of cortical bone in the femoral neck and hip fracture: A prospective case-control analysis of 143 incident hip fractures; the AGES-Reykjavik Study. Bone.

[33] Xu Y., Zhu Y., Lin W., Lee D.H., Yang F., Fan Y. (2024). Quantitative computed tomography analysis of proximal femur bone mineral density and its relation to hip fracture risk. Quant. Imaging Med. Surg..

[34] Lee S.-W., Yoon Y., Kwon J., Heu J.-Y., Hwang J. (2023). Clinical significance of discordance between hip and spine bone mineral density in Korean elderly patients with hip fractures. J. Clin. Med..

[35] Leslie W.D., Lix L.M. (2010). Simplified 10-year absolute fracture risk assessment: A comparison of men and women. J. Clin. Densitom..

[36] Kanis J.A., Johnell O., Oden A., Johansson H., McCloskey E. (2008). FRAX and the assessment of fracture probability in men and women from the UK. Osteoporos. Int..

[37] Fan B., Lu Y., Genant H., Fuerst T., Shepherd J. (2010). Does standardized BMD still remove differences between Hologic and GE-Lunar state-of-the-art DXA systems?. Osteoporos. Int..

